# A polymeric hydrogel electrocatalyst for direct water oxidation

**DOI:** 10.1038/s41467-023-36532-x

**Published:** 2023-02-13

**Authors:** Zengxia Pei, Hao Tan, Jinxing Gu, Linguo Lu, Xin Zeng, Tianqi Zhang, Cheng Wang, Luyao Ding, Patrick J. Cullen, Zhongfang Chen, Shenlong Zhao

**Affiliations:** 1 School of Chemical and Biomolecular Engineering, The University of Sydney, Sydney, NSW 2008 Australia; 2 National Synchrotron Radiation Laboratory, University of Science and Technology of China, Hefei, 230029 PR China; 3 Department of Chemistry, University of Puerto Rico, Rio Piedras Campus, San Juan, PR USA

**Keywords:** Electrocatalysis, Electrocatalysis

## Abstract

Metal-free electrocatalysts represent a main branch of active materials for oxygen evolution reaction (OER), but they excessively rely on functionalized conjugated carbon materials, which substantially restricts the screening of potential efficient carbonaceous electrocatalysts. Herein, we demonstrate that a mesostructured polyacrylate hydrogel can afford an unexpected and exceptional OER activity – on par with that of benchmark IrO_2_ catalyst in alkaline electrolyte, together with a high durability and good adaptability in various pH environments. Combined theoretical and electrokinetic studies reveal that the positively charged carbon atoms within the carboxylate units are intrinsically active toward OER, and spectroscopic operando characterizations also identify the fingerprint superoxide intermediate generated on the polymeric hydrogel backbone. This work expands the scope of metal-free materials for OER by providing a new class of polymeric hydrogel electrocatalysts with huge extension potentials.

## Abstract (web summary)

Hydrogels are networked hydrophilic polymers with enriched polar groups and confined water but are relatively less explored as electrocatalysts. Here, the authors demonstrate that insulative polymeric hydrogels can be an underlying catalogue of metal-free oxygen evolution electrocatalyst with huge extension potentials.

## Introduction

Electrocatalytic OER is essential in many key energy conversion and storage systems, especially for water splitting electrolyzers and rechargeable metal-air batteries^1–5^. The OER involves multi-electron and multi-intermediates, thus pertinent electrocatalysts are imperative to expedite the reaction kinetics. Carbonaceous metal-free electrocatalysts have been vibrantly exploited as an important class of OER catalysts complementary to metal-based ones in recent years^5–7^. Heteroatom doping^5,8–10^, defect engineering^11,12^, and intermolecular functionalization of carbon substrates (e.g., carbon nanotube, graphene)^13,14^ are typical synthetic strategies towards such metal-free OER electrocatalysts. Essentially, all these strategies induce the charge redistribution on conjugated carbon matrix, thereby some of the locally positively charged (or termed as p-type domain) carbon atoms are activated to serve as active sites for OER. In other words, the key is to induce positively charged carbon, which is indispensable for the elementary adsorption of hydroxide intermediates during OER^5,8,10,14^. However, relying on carbon allotropes as source materials restricts the choice of precursors, whilst most of the aforementioned modification approaches also involve complicated and/or energy-consuming procedures (e.g., calcination). Hence, it is highly desirable, yet challenging, to search for easily fabricated efficient metal-free OER electrocatalysts beyond carbon.

Hydrogels are a class of hydrophilic polymers with three-dimensional (3D) networks, which contain repeated polar group (e.g., carboxyl group) units to confine a high content of water/electrolyte^15^. Most hydrogels are easily fabricated from cheap precursors under mild conditions, while their 3D network avoids the dissolution of the polymer chains (this ensures heterogeneous electrocatalytic reactions with excellent processability). More significantly, the polarized groups render enriched positive carbon atoms within the hydrogel skeleton, which can be potential OER active sites^15,16^. Besides, the confined gel–water/electrolyte species have been demonstrated to have intriguing interactions with the polymer skeletons, thereby leading to enhanced electrocatalytic activities^17–20^. In this context, due to the huge diversity of the polymer types and its large tunability, polymeric hydrogels may represent an underlying catalogue of metal-free OER electrocatalysts with great extension potentials. However, hydrogel-based metal-free OER electrocatalysts have been rarely reported so far.

Herein, we demonstrate that a mesostructured polyacrylate (PANa) hydrogel can be an efficient and robust OER electrocatalyst on various conductive substrates (e.g., carbon cloth (CC), copper foam (CuF), gold electrode (AuE)) over a wide pH range (0–14). The PANa hydrogel is chosen because of its high theoretic density of positively charged carbon atoms (1 out of every 3) in the polymer chain, representing an ideal scaffold material for OER. Combined density functional theory (DFT) calculations and operando characterization validate that the OER activity indeed stems from the positively charged carbon atom within the carboxylate group. As expected, the optimal PANa based electrode afford a metric OER current density of 10 mA cm^−2^ at an overpotential of 316 mV - on par with the benchmark IrO_2_, together with a small Tafel slope of 42 mV dec^−1^ and an excellent durability.

## Results

### Theoretical study of the hydrogel electrocatalyst

**Fig. 1:**
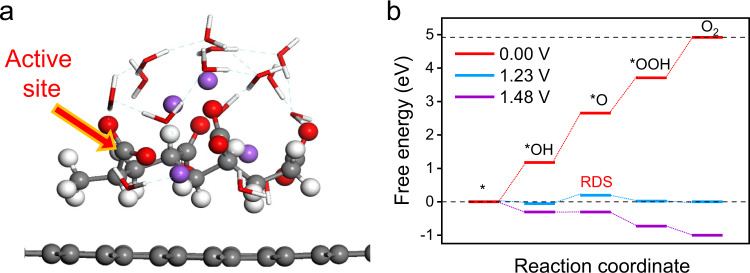
Theoretical prediction of the OER activity of PANa. **a** Optimized structure of PANa molecule with graphene substrate, with one typical positive C atom of the polymer chain indicated by an arrow. Gray, red, purple, and white balls represented carbon, oxygen, sodium, and hydrogen atoms, respectively. **b** Free energy diagram of the positive C in PANa for OER.

To explore the feasibility of the PANa hydrogel as an OER electrocatalyst, DFT calculations were first performed on the PANa molecules. Charge analysis suggests that, among all the carbon atoms within the hydrogel chains, only those in carboxylate units feature an average charge of +0.195 e (Supplementary Fig. 1), comparable to those in heteroatom-doped carbon materials. It is well established that the positively charged carbon atoms can accept electrons from OH^−^ to form *OH, which is the elementary step of OER^1,5^. As such, the OER activity of the positive carbons within PANa hydrogel moieties was examined by plotting the free energy diagrams under specified potentials, with a carbon sheet as a conductive substrate (Fig. 1a). As shown in Fig. 1b, at the theoretical potential of 1.23 V, all OER steps were thermodynamically favourable except the formation of ^*^O, suggesting the second step (step II, i.e., *OH → *O) is the rate-determining step (RDS) for OER. This step then becomes favourable upon increasing the potential to 1.48 V, indicating that the supported PANa has an unexpected small overpotential of 250 mV for OER. For bare PANa hydrogel (without a conductive substrate), a moderate overpotential of ca. 390 mV will be mandatory for catalyzing OER (Supplementary Figs. 2–5), with the initial adsorption of *OH (step I) being the RDS. The larger required overpotential here can be understood that the interfacial charge redistribution with the substrate can effectively boost OER kinetics of the PANa hydrogel (Supplementary Fig. 6). Note all DFT calculations were performed in a water environment because hydrogen bonding of the surrounding water plays significant roles in modulating the reaction intermediates^21,22^. This can also better illustrate the real electrocatalytic OER process and reflect the hydrogel nature of PANa material (Supplementary Fig. 7). The detailed reaction pathways and binding configurations along the optimal OER pathway on the graphene-supported positive carbon within carboxylate units are presented in Supplementary Fig. 8. In summary, our DFT calculations suggest that the PANa hydrogel can theoretically be a very active OER electrocatalyst.

### Synthesis and characterization of the hydrogel composite electrode

**Fig. 2:**
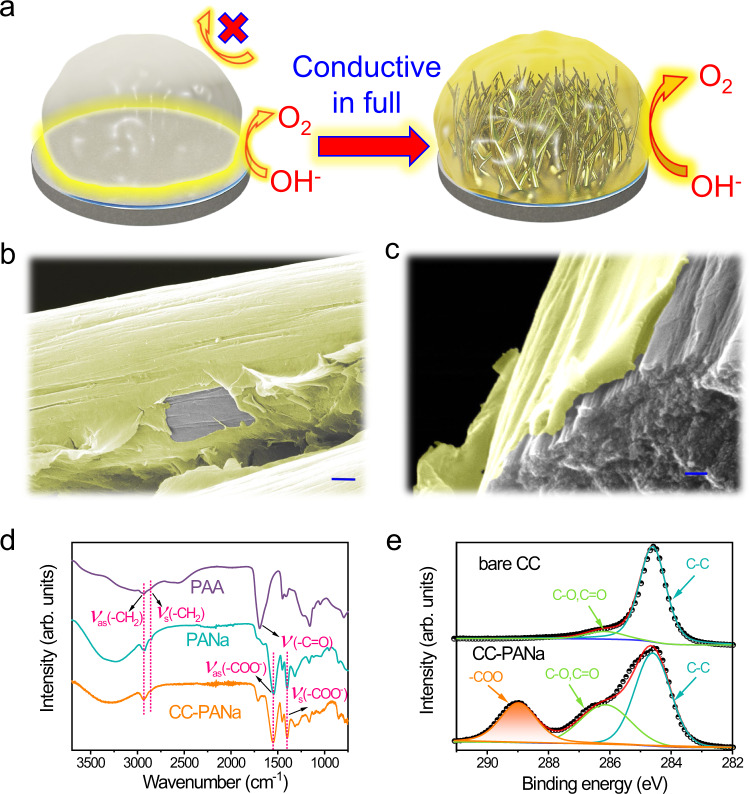
The morphology and structure characterization of the CC-PANa electrode. **a** Schematic diagram showing a hierarchical conductive substrate can fully activate the insulative PANa hydrogel for more efficient OER, with the yellow areas represent active regions for OER. **b, c** SEM images showing the CC-PANa electrode, where the yellow layer is the PANa moiety. **d** ATR-IR spectra of PANa hydrogels and CC-PANa electrode, with un-alkalized PAA hydrogel as a reference. **e** C 1*s* XPS spectra of bare CC and CC-PANa electrode. Scale bars: **b** 1 μm, **c** 200 nm.

Inspired by the computational results, we synthesized a mesostructured PANa hydrogel coupled onto various conductive substrates using an in situ dip coating-polymerization method (detailed procedures are given in the experimental section, and illustrated in Supplementary Fig. 9). A 3D hierarchical conductive substrate, like CC and CuF, is preferential since it can better activate the whole skeleton of PANa hydrogel rather than the restricted local area on the flat hydrogel-substrate interface (illustrated in Fig. 2a). Note that the concentration of the initial monomer solution can be adjusted to render an appropriate amount of PANa hydrogel on the substrates (Supplementary Figs. 10 and 11). Scanning electron microscopy (SEM) images show that, on the typical surface of CC, the thickness of the well-encapsulated hydrogel is around 50 nm, with more or fewer precursors leading to overwrapping or incomplete patching on the carbon fibers, respectively (Fig. 2b, c, Supplementary Fig. 10). As can be envisaged, the barren coverage of PANa on CC will render limited OER activity, while an excessive amount of the hydrogel blocks the diffusion of both electrolyte and evolved oxygen gas. Note here the PANa hydrogel was directly dried for easy determination of its thickness, whilst it actually possesses typical porous structure as shown in the freeze-dried sample (Supplementary Fig. 12). Such porous structure can facilitate the release of the evolved O_2_ gas ^23,24^.

The attenuated total reflectance infrared (ATR-IR) spectrum of the PANa-beared CC (CC-PANa) electrode displays two strong characteristic peaks at 1545 and 1400 cm^−1^, assigned to the asymmetric and symmetric stretching bands of carboxylate (–COO^−^) group, respectively^25^. Another intermediate peak at 1710 cm^−1^ stems from the vibration mode of residual carboxyl groups^26^. The other weak doublet peaks at around 2930 and 2860 cm^−1^, which are also observed in the referenced bare PANa and PAA hydrogels, are caused by the stretching of the methylene moiety within the allyl backbones^26^. These results suggest the successful incorporation of PANa hydrogel onto the CC substrate. As expected, X-ray photoelectron spectroscopy (XPS) reveals that after PANa coating, an intense signal from the –COO^−^ groups is observed in the C 1*s* core level of CC-PANa electrode, indicating substantially enriched active sites for OER compared with that in the bare CC. Moreover, the CC-PANa electrode surface is superhydrophilic (Supplementary Fig. 13, Supplementary Videos 1 and 2), which can facilitate the transport of electrolytes to expedite OER kinetics.

### Electrocatalytic measurements for water oxidation

**Fig. 3:**
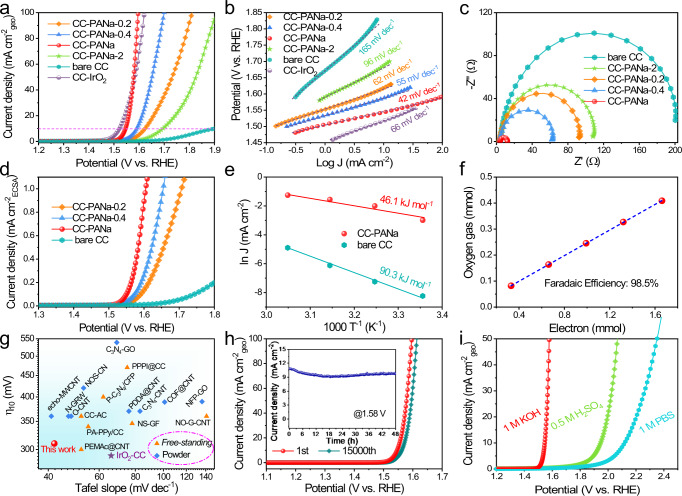
Electrocatalytic OER performance of the CC-PANa electrodes. **a** Polarization curves and **b** Tafel slopes of different electrodes. **c** EIS spectra. **d** Normalized (by ECSA) OER current density of the electrodes. **e** Arrhenius plots with the activation energies listed. **f** Comparison of evolved O_2_ vs. the amount of consumed electrons during the course of electrolysis. **g** Comparison of the OER performance of different electrodes/materials. **h** Polarization curves before and after stability scans, inset shows the i-t profile. **i** OER activity of the CC-PANa electrode in different electrolytes.

The electrochemical performance of the hydrogel-modified electrodes toward OER was first evaluated in O_2_-saturated 1 M KOH solution, with all potentials calibrated to the reversible hydrogen electrode (RHE, Supplementary Fig. 14). The working electrodes were also coated with epoxy glue to avoid the capillary rise of the electrolyte (Supplementary Figs. 15 and 16)^27,28^. As shown in Fig. 3a, the CC-PANa electrode affords a metric overpotential at 10 mA cm^−2^ (η_10_) of 316 mV, which is not only dramatically smaller than that of the bare CC (640 mV) but also approaching that of the benchmark IrO_2_ (289 mV). Notably, the Tafel slope of the CC-PANa electrode is only 42 mV dec^−1^ (even smaller than the corresponding value of 66 mV dec^−1^ for IrO_2_, Fig. 3b), enabling a large OER current density of 100 mA cm^−2^ only at 1.596 V (vs. RHE). For OER in alkaline electrolytes, it is generally accepted that the Tafel slope for step I and step II is about 120 and 40 mV dec^−1^, respectively^10,29^. The small Tafel slope here indicates the RDS is step II for the CC-PANa electrode, which coincides well with the DFT calculations (Fig. 1b). By contrast, the bare CC shows a large Tafel slope of 165 mV dec^−1^, implying lacking polar active sites is the main barrier for the elementary OH^−^ adsorption (see discussions in Supplementary information (SI)).

The OER activity of the electrode is dependent on the PANa content. The initial gradual increase of the precursor concentration leads to larger coverage of the PANa, thus the increased density of polar –COO^−^ groups (on the otherwise inactive carbon fiber surface) can enhance OER activity (as shown by the activity of CC-PANa-0.2 to CC-PANa, Fig. 3a, b, Supplementary Fig. 17). However, overwrapping of the hydrogel in the CC-PANa-2 electrode will hinder the transport of electrolyte and evolved gas, incurring a much larger η_10_ and Tafel slope of 458 mV and 96 mV dec^−1^, respectively. Such a conclusion is also validated by the electrochemical impedance spectroscopy (EIS) analyses (Fig. 3c), where the CC-PANa electrode delivers the smallest charge transfer resistance. To reveal the contribution from the polar active sites, we normalized the OER current density of the series electrodes by their electrochemical surface areas (ECSAs) (Supplementary Fig. 18). As shown in Fig. 3d, the normalized OER activity (by ECSA) is still positively correlated with the PANa gel content, attesting the active site should be the polar –COO^-^ groups. Such a conclusion was further verified by control experiments using different content of crosslinker and other polymers with tuneable concentration of carboxylate group (Supplementary Figs. 19–21). The possible contribution of metal ions was excluded by several combined control experiments (including inductively coupled plasma atomic emission spectroscopy (ICP-AES), XPS, and poisoning test, see Supplementary Figs. 22–25, Supplementary Table 1), further validating the critical role of the carboxylate groups within PANa hydrogel toward OER.

To specifically evaluate the kinetic energy barriers involved in OER, the apparent activation energy (*E*_a_) of the CC-PANa and the bare CC electrodes was compared. As shown in Fig. 3e, at a typical overpotential of 350 mV, the CC-PANa electrode features an *E*_a_ of 46.1 kJ mol^−1^, almost half of that of the bare CC. Note that at elevated temperatures, both electrodes show enhanced OER performance with reduced onset potentials (Supplementary Fig. 26), indicating a similar reaction mechanism for them. Assuming the identical active sites, the smaller *E*_a_ unravels the introduced abundant –COO^−^ groups on CC can indeed substantially diminish the energy barrier for OER. The Faradaic efficiency measurement further reveals a 98.5% efficiency of the CC-PANa electrode toward OER without any detectable impurities (Fig. 3f, Supplementary Fig. 27). The turnover frequency of the optimized PANa was calculated to be 1.65 × 10^−2^ s^−1^ at an overpotential of 350 mV, comparable to (within the same order of magnitude) that of IrO_2_ (5.18 × 10^−2^ s^−1^), highlight the hydrogel’s intrinsic activity for OER (see discussions in SI). Actually, the CC-PANa electrode outperforms most of recently reported metal-free electrocatalysts (of both powder and free-standing forms) in terms of the overall activity (Fig. 3g, Supplementary Table 2). Though the CC-PANa cannot rival state-of-the-art transitional metal-based OER electrocatalysts (e.g., Ni-FeOOH that has an η_10_ ≤ 200 mV), the great tunability (given the diversity of polymer types), and simple and green synthetic methods still make this type of polymeric hydrogel electrocatalysts have its own advantages. Of equal importance, the composite electrode affords good durability for 48 h of chronoamperometric (CA) test and 15,000 cyclic scans (Fig. 3h). The slight current decrease could be caused by the inevitable partial detachment of the hydrogel from the conductive substrate, since the hydrogel will swell in the electrolyte and the gas-evolving process will also exacerbate the partial detachment. Such assumption was then corroborated by the post-OER SEM observations and mechanical test of the CC-PANa electrode (Supplementary Figs. 28 and 29). Nevertheless, the chemical structure of the gel did not show obvious changes as observed from the XPS and ATR-IR spectra (Supplementary Fig. 30). The CC-PANa electrode actually delivers much better OER stability than the IrO_2_-based counterpart (Supplementary Fig. 31). Further, the CC-PANa electrode delivers decent OER activity in both acidic and neutral phosphate buffer solution electrolytes (Fig. 3i), highlighting its intrinsic adaptability in various pH environments (Supplementary Fig. 32). However, the electrodes show more serious activity decay in acidic and neutral electrolytes compared with the case in alkaline one (Fig. 3h), because the overpotentials required for OER in non-alkaline electrolytes are too high, and the acidic electrolyte is highly corrosive. Hence, while the CC-PANa electrode do exhibit activity in different electrolytes, it is more suitable for OER in alkaline ones. Moreover, the flexible nature of the electrode allows it to work under repeated deformations (Supplementary Fig. 33), manifesting its good processability. Note that the PANa gel can be easily coated onto other substrates, such as CuF, AuE, and glassy carbon electrode, to work efficiently as OER catalysts (Supplementary Figs. 34 and 35), which not only verifies the inherent OER active sites in the hydrogel, but also shows the great potential for possible scale-up applications.

### Spectroscopic verification of the active sites and intermediate

**Fig. 4:**
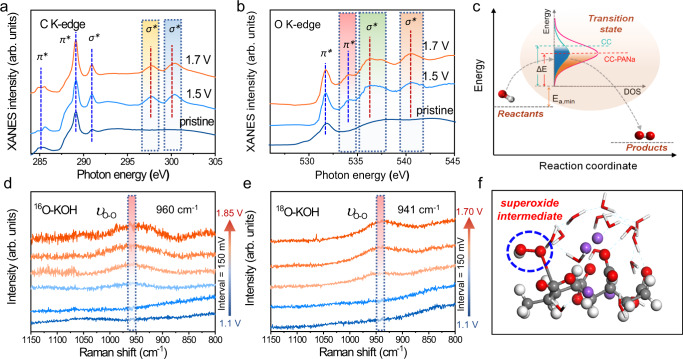
The active sites and superoxide intermediate during OER. High-resolution XANES spectra of **a** C K-edge and **b** O K-edge record from the PANa gel before and after chronoamperometric OER tests at 1.5 and 1.7 V (vs. RHE), with the unique resonances in post-OER sample highlighted in coloured rectangles. **c** Energetic illustration of the OER process on the electrocatalysts, the highlighted region represents the transition states, *E*_a,min_ is the minimum activation energy required and Δ*E* is the overpotential. In operando Raman spectra of the gel electrocatalyst under various applied potentials (vs. RHE) in **d** 1 M ^16^O-KOH and **e** ^18^O-KOH electrolytes, the coloured bars highlight the stretching vibration of *O-O intermediate. **f** Schematic showing the *O-O intermediate adsorbed on the C atom within a carboxylate group.

We also performed X-ray absorption near-edge structure (XANES) analyses to check the gel electrocatalyst. Figure 4a shows the C K-edge spectra of the PANa gel before and after OER. The peaks at ca. 285.2 and 290.9 eV are caused by the 1*s* − *π* * and 1*s* − *σ* * transitions in C–C bond in the gel backbone, respectively^5,30,31^. Another characteristic sharp peak at 288.9 eV is attributed to 1 *s* − *π* * transition of the –COO^-^ moiety^32,33^. All these three peaks were preserved after the CA test, suggesting the good stability of the gel electrocatalyst during OER. The other two peaks at 297.8 and 300.4 eV, observed exclusively in the post-OER gels, can be assigned to the 1 *s* - *σ* * excitation of –COO^−^^31,32^. Similarly, the O K-edge spectra in Fig. 4b reveal the peak at 531.7 eV (1 *s* − *π* * excitation of –COO^−^) appeared in all samples, while the two doublet peaks at 536.3 and 540. 4 eV (1 *s* − *σ* * excitation of –COO^−^) emerged only in the samples after OER^32,34,35^. These results validate the –COO^−^ group is intensely excited during OER, probably via acting as the active site. Such a conclusion is further evidenced by the enhanced 1 *s* - *π* * excitation (the peak at 534.1 eV) resulting from the charge transfer between C and O in –COO^−^ group^32,34,35^. In other words, the applied anodic potential intensifies this charge transfer progress, thus rendering the C site more positive charge to facilitate the elementary adsorption of OH^−^ during OER. The XANES analyses therefore coincide well with the DFT calculations, both confirming that the polar –COO^−^ groups can catalyze OER. As illustrated in Fig. 4c, the reactants need to overcome certain activation barriers (*E*_a,min_ + Δ*E*) to form the transition state and finally to form the products. The abundant –COO^−^ groups of PANa, with their multiplet transitions, essentially impart more reactive density of states (DOS) compared with the relatively inert substrate (e.g., CC). During OER, the active sites in CC-PANa with higher activity (hence a smaller activation energy, Fig. 3e) and deeper DOS distribution will be more easily occupied by OH^-^, forming the transition state till the eventual liberation of O_2_.

As a further consolidation of the catalytic effect of the –COO^−^ group, in operando Raman spectroscopy was performed to investigate the adsorption behaviours of possible OER intermediates (Fig. 4d–f). In particular, the characteristic vibration of the superoxide intermediate (*O–O) has been mostly detected by in operando Raman spectroscopy to provide fingerprint information for OER^36–38^. As such, we first recorded the Raman spectra by stepwise varying the anodic potential in O_2_-saturated 1 M ^16^O-KOH solution. Figure 4d shows that a broad band centred at 960 cm^−1^ gradually emerged when the potential is above 1.4 V (vs. RHE). Our DFT calculation result (Supplementary Table 3) suggests the adsorbed *O–O on the C atom within the –COO^-^ group will generate a stretching vibration mode at 998 cm^−1^. The observed peak here thus can be safely assigned to the *O-O during OER (illustrated in Fig. 4f). The minor difference between the theoretical and experimental values might reasonably stem from the influence of the solvent molecules in realistic reaction environment. More critically, when replacing the ^16^O-KOH with ^18^O-KOH, the observed band still retained but red-shifted for 19 cm^−1^ (Fig. 4e), which is close to the calculation result (951 cm^−1^, Supplementary Table 3) and is similar to previous reported trends based on metal compounds^36,37^. Therefore, the in operando Raman spectroscopy and the isotope labelling results firmly verify that the featuring *O-O species is generated on the –COO^−^ group during OER. To the best of our knowledge, this is the first spectroscopic evidence of the key OER intermediate in metal-free electrocatalysts.

### Generality of the polymeric hydrogel OER electrocatalyst

**Fig. 5:**
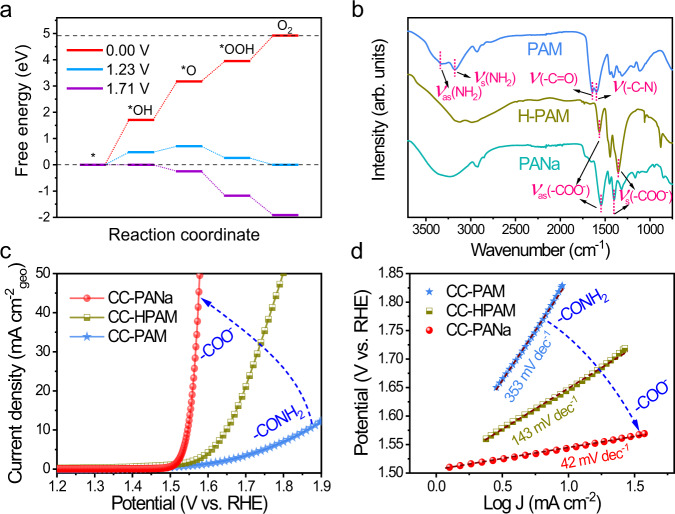
Verification of the OER-active sites in hydrogels. **a** Free energy diagram of the positive C in PAM for OER. **b** ATR-IR spectra of different hydrogels. **c** Polarization curves and **d** Tafel slopes of the electrodes.

To review the generality of the polymeric hydrogel materials as OER electrocatalysts, we also studied the OER activity of another most common allyl polymer, polyacrylamide (PAM). The PAM hydrogel shares a very similar chemical structure to that of PANa, except for the different terminal groups (Supplementary Figs. 1, 36). However, DFT calculations suggest that the PAM hydrogel requires a much larger overpotential of 480 mV for OER, presumably due to the less positive charge of the carbon atom (+0.152 e, the only active site) in the terminal amide group (Fig. 5a, Supplementary Figs. 36, 37). These results suggest adequately positive carbon sites are necessary for polymeric hydrogel as OER electrocatalysts, though the optimal charge density still needs further investigation. It is well-known that the PAM can be partially hydrolyzed into a co-polymer of PAM-PAA (or PAM-PANa in alkaline, Supplementary Fig. 38) at elevated temperatures^39,40^, which provides an ideal approach to compare the intrinsic activity of the two hydrogels. As such, we hydrolyzed the PAM (kept at 70 °C for 1 h in 6 M KOH, marked as HPAM,). ATR-IR spectra in Fig. 5b reveal the evolution of the characteristic peaks from –CONH_2_ groups into –COO^−^ ones in the HPAM sample. Interestingly, the CC-HPAM electrode affords much improved OER activity compared with that of the pristine CC-PAM counterpart (Fig. 5c, d). The gradually enhanced activity provides further evidence proving that the –COO^−^ group is a more active site for OER, which also suggests the huge potential of developing efficient hydrogel-based OER electrocatalysts via tuning the polymer chemistries.

## Discussion

Through DFT calculations, electrokinetic tests, and in operando spectroscopic measurements, we demonstrated the positively charged carbon atoms within the PANa hydrogel are intrinsically active toward OER. The mesostructured PANa hydrogel displayed an excellent OER activity (with an η_10_ of 316 mV and a Tafel slope of 42 mV dec^−1^) on par with the noble metal electrocatalyst (e.g., IrO_2_) in alkaline electrolyte, together with a good durability, and remarkable adaptability in various pH environments. The fingerprint superoxide intermediate was identified for the first time among all reported metal-free OER electrocatalysts. The fabrication of the composite electrode is green, pyrolysis-free, binder-free, and cost-effective, which holds great potential for scale-up. The results of this work underpin the connotation of metal-free electrocatalysts by providing a new class of polymeric hydrogel OER materials with huge extension potentials. The design strategies are expected to inspire further development of various metal-free electrocatalysts for other catalytic reactions and applications.

## Methods

### Materials

Acrylic acid (AA, ≥99.0%), ammonium persulfate ((NH_4_)_2_S_2_O_8_, ≥98.0%), N, N′-methylenebisacrylamide (MBAA, ≥ 99.5%), potassium hydroxide (KOH, ≥ 99.99%), potassium thiocyanate (KSCN, ≥ 99.0%), H_2_^18^O (97% isotopic purity) were purchased from Sigma-Aldrich. Sodium hydroxide (NaOH, ≥99.9%) was purchased from Aladdin. Carbon cloth (CC) was purchased from CeTech Co., Ltd. Copper foam (CuF) was purchased from Sigma-Aldrich. Deionized water purified by a Milli-Q system was used throughout the experiments.

### Electrode synthesis

Before fabricating the CC-PANa electrodes, the CC was first calcinated at 300 °C for 1 h in air and washed with 6 M HCl (instead of oxidative HNO_3_ to avoid introducing too much polar groups onto the carbon fiber surface), followed by repeated thorough washing with DI water. The calcination followed by acid and water washing can effectively remove the possible metal impurities but ensure the CC surface is relatively inert.

The CC-PANa series electrodes were synthesized by an in situ dip-coating-polymerization method. Typically, 3.5 mL AA monomer was dissolved in 13.5 mL of water, and then a concentrated NaOH solution (5 mL, 10 M) was added dropwise into the AA solution under stirring at 0 °C. Prior to use, the AA monomer was purified by distillation under reduced pressure and stored in a refrigerator. Afterward, 0.1 wt% of MBAA was added as a chemical cross-linker, followed by the addition of 110 mg (NH_4_)_2_S_2_O_8_ (as initiator). This matrix solution was sonicated for 5 min, stirred for 15 min, and stored at 0 °C for further use.

To prepare the CC-PANa electrode, one piece of the pre-cleaned CC (0.5 × 4 cm) was directly dipped into the above matrix solution for about 3 min, taken out with careful wipe, and was then subject to free-radical polymerization at 65 °C for 2 h (illustrated in Supplementary Fig. 9). To control the amount of coated PANa hydrogel, the above matrix solution was diluted 2.5 or 5 times as much as the original by water, followed by the above dip-coating-polymerization treatment. The resulting electrodes were marked as CC-PANa-0.4 and CC-PANa-0.2, respectively. For CC-PANa-2 electrode, the initial volume of AA and water was 7 and 10 mL (i.e., doubling the AA precursor concentration), respectively, with all other procedures remained identical. For the optimal CC-PANa electrode in this work, the loading mass of PANa part was 0.8 ± 0.2 mg cm^−2^ (based on geometric area of the CC substrate, determined by a semi-micro balance) from batch to batch. As a reference, commercial IrO_2_ was drop-casted (using Nafion solution as binder) onto the CC substrate with an effective loading mass of 0.6 mg cm^−2^.

The PANa-loaded CuF electrodes were synthesized similar to the CC-PANa electrode except for using pre-cleaned CuF as substrates. To prepare the planar electrodes, 2 uL of the matrix solution was drop cast onto the mirror-polished glassy carbon electrode (GCE) and golden disk electrode (AuE), followed by drying at 65 °C for 2 h.

### Physicochemical characterizations

The morphology and microstructure of samples were examined by a Hitachi field-emission SEM. ATR-IR spectra were collected on the Spectrum 100 spectrometer with an ATR-IR signal collector. XPS analyses were conducted on a Thermo Fisher ESCALAB 250 photoelectron spectrometer at 1.2 × 10^−9^ mbar using an Al K_α_ X-ray beam (1486.6 eV). XPS spectra were charge corrected to the adventitious C 1 s peak at 284.6 eV. The C and O K-edge XANES spectra were collected at the BL12B beamlines of National Synchrotron Radiation Laboratory (Hefei, China). In operando Raman spectra were recorded using a LabRam HR Raman Spectrometer, with a laser wavelength of 532 nm.

### Electrochemical measurements

All electrochemical measurements were carried out on a CHI 760D electrochemical workstation in a typical 3-electrode system. The free-standing CC-PANa electrode or the CuF-PANa electrode served as the working electrode, an Ag/AgCl (filled with saturated KCl) electrode and a Pt mesh were used as the reference and counter electrode, respectively. For these porous electrodes, an epoxy glue was used to coat them to avoid the capillary rise of the electrolyte, and the effective reaction area was about 0.5 × 1.0 cm. For planar working electrodes, the catalyst-bearing GCE or AuE (diameter 5 mm) was integrated with a rotating disk electrode apparatus (Pine Electrochemical set, USA), with a rotation speed of 1600 rpm. 1 M KOH, 0.5 M H_2_SO_4_, and 1 M phosphate buffer saline (PBS) were used as the alkaline, acidic, and neutral electrolytes, respectively. Prior to all measurements, the electrolyte was bubbled with O_2_ for 30 min. The temperature of the reactor cell was controlled by a circulating water tank at desired temperatures. The recorded potentials were converted to RHE by calibration (details see Supplementary Fig. 14). The polarization curves were obtained at a scan rate of 5 mV s^−1^. All the curves were iR-corrected unless otherwise stated. The electrode was initially swept for a few cycles to get a stable current. The Tafel slopes were recorded at 1 mV s^−1^. For stability evaluation, the chronoamperometric test was conducted at 1.58 V (vs. RHE). The accelerated degradation test (ADT) was conducted by cycling different electrodes in a potential range of 1.2–1.7 V (vs. RHE) at 100 mV s^−1^, without iR correction.

The Faradaic efficiency of the electrode was evaluated via a volumetric method^5^. In detail, the produced O_2_ over the CC-PANa electrode (with an effective area of 1.0 cm^−2^) was accumulated in a graduated tube, which was filled with the electrolyte. Current dominated electrolysis was executed at 10 mA cm^−2^ under ambient conditions (22°C, 1 atm). The time was recorded at every 2 ml of O_2_. The collected charges going through the working electrode, in the meantime, were reckoned via current × time. The collected gas (on the top of the graduated tube) was sampled with a Hamilton syringe and detected by using a gas chromatograph (Shimadzu GC2030) to evaluate its purity, which shows the O_2_ is the only gaseous product without other impurities.

### Computational methods

All spin-unrestricted density functional theory (DFT) calculations were carried out using DMol^3^ package^41^. To simplify the modelling of polyacrylate chain, the molecule containing four repeating acrylate units was simulated. The chemical formula of the molecule can be marked as H-[CH_2_CHCOONa]_4_-H. No periodic boundary conditions were used to examine the OER performance of the molecule. Graphene supported polyacrylate was simulated by placing the molecule on top of a graphene monolayer. Here, the periodic boundary conditions were used to examine the OER performance of the graphene supported molecule. The polyacrylamide models were constructed in a similar way. The Perdew–Burke–Ernzerhof (PBE) functional within the generalized gradient approximation (GGA)^42^ was employed. We also considered van der Waals interactions following the empirical correction in Grimme’s scheme^43^. The solvent effects were treated using the hybrid (or cluster-continuum) approach which combines explicit and implicit solvation models and thus brings together key advantages of both models^44^. For the implicit conductor-like screen model (COSMO), the dielectric constant for water is 78.54^45^. For the explicit solvent model, 16 water molecules were used as the core solvation shell. The details for hybrid solvent model are given in SI. The double numerical plus polarization (DNP) basis set was utilized^46^. A global orbital cutoff of 3.5 Å, an energy convergence level of 2.0 × 10^−5^ Ha, and an SCF tolerance of 1.0 × 10^−5^ Ha were adopted. Atomic charge analyses were performed by the Hirshfeld method^47^. The Gibbs reaction free energy change (∆*G*) of each elementary step was evaluated based on the computational hydrogen electrode (CHE) model developed by Nørskov and coworkers^48^. The free energy *G* for each species is

$$G=E+{E}_{{{\mathrm{ZPE}}}}-{TS}$$

where *E* is the total energy, *E*_ZPE_ is the zero-point energy, *S* is the entropy, and *T* is set to 300 K. For all the species, the total energies are obtained by DFT computations. The *E*_ZPE_ and *S* values of O-containing intermediates (*O, *OH, *OOH), H_2_O, and H_2_ molecules are summarized in Supplementary Table 4.

## Supplementary information

Supplementary information

Description of Additional Supplementary Files

Supplementary Video 1

Supplementary Video 2

## Data Availability

The source data underlying Figs. 1–5 generated in this study is provided as a Source Data file. Additional data are available from the corresponding authors upon reasonable request. Source data are provided with this paper.

## Source data

Source Data
