# Supplementary material for: A polymeric hydrogel electrocatalyst for direct water oxidation
Source: Nat Commun. 2023 Feb 13;14:818. doi: 10.1038/s41467-023-36532-x (PMC9925792; doi:10.1038/s41467-023-36532-x)
Supplement: Supplementary file 1 — Supplementary information [file 41467_2023_36532_MOESM1_ESM.pdf]

Supplementary Information for

A Polymeric Hydrogel Electrocatalyst for Direct Water Oxidation

Zengxia Pei,[1,#,*] Hao Tan,[2,#] Jinxing Gu,[3,#] Linguo Lu,[3] Xin Zeng,[1] Tianqi Zhang,[1] Cheng Wang,[1] Luyao Ding,[1] Patrick J. Cullen,[1] Zhongfang Chen,[3,*] Shenlong Zhao[1,*]

[1] School of Chemical and Biomolecular Engineering, The University of Sydney, Sydney, New South Wales 2008, Australia

[2] National Synchrotron Radiation Laboratory, University of Science and Technology of China, Hefei 230029, P. R. China

[3] Department of Chemistry, University of Puerto Rico, Rio Piedras Campus, San Juan, Puerto Rico, United States

*Corresponding authors: zengxia.pei@sydney.edu.au (Z. Pei)

zhongfangchen@gmail.com (Z. Chen)

shenlong.zhao@sydney.edu.au (S. Zhao)

[#] These authors contributed equally to this work.

Calculations

***Calculation of OER rate-determing step (RDS)**:* a well-established mechanism for OER in alkaline media can be expressed as following:[1, 2]

$* + OH^- \leftrightarrow *OH + e^-$ (I)

$*OH + OH^- \leftrightarrow *O + H_2O + e^-$ (II)

$*O + OH^- \leftrightarrow *OOH + e^-$ (III)

$*OOH + OH^- \leftrightarrow * + O_2 + H_2O + e^-$ (IV)

Where * refers to the adsorption site of an electrocatalyst.

Note the Tafel test in this study has been carried out at a low scan rate (1 mV s^{-1}), hence it can be assumed that only the RDS is irreversible while other steps are in the quasi-equilibrium state. Given step (I) is the DRS, the Tafel slope can be expressed by:[1, 2]

$$\frac{\partial\eta}{\partial\log(i)} = \frac{\partial\eta}{\partial\log(v)} = \frac{2.3RT}{\beta F} = b$$

If step (II) is the RDS, then the Tafel slope can be expressed by:

$$\frac{\partial\eta}{\partial\log(i)} = \frac{\partial\eta}{\partial\log(v)} = \frac{2.3RT}{(1+\beta)F} = b$$

Where η is the applied overpotential, i is the current density, v is the reaction rate, and b is the Tafel slope. β is the symmetric factor, referring to the fraction of the overpotential goes towards lowering the activation barrier for the reaction. R is the gas constant, T is the temperature (K), F is the Faradaic constant.

β essentially depicts the influence overpotential on the activation barries, and can be obtained from the partial derivative of the Marcus equation: [1, 2]

$$\beta = \frac{\partial G_f^*}{\partial\eta} = \frac{1}{2} + \frac{\eta}{2\lambda}$$

Where ∂G_f^* (eV) and λ (eV) are the activeation barrier and reorgainzation energy, respectively. For most systems at room temperature (293.15 K), λ is much larger than 1 eV, hence $\frac{\eta}{2\lambda}$ becomes insignificant and β approaches 0.5. Therefore, at room temperature, Tafel slope with step (I) and step (II) as RDS is 120 and 40 mV dec^{-1}, respectively.

In this study, the Tafel of the CC-PANa sample is 42 mV dec^{-1}, which is very close to 40 mV dec^{-1}, suggesting step (II) is the RDS. By contrast, the bare CC electrode shows a Tafel slope of 165 mV dec^{-1}, implying lacking polar active sites is the main barrier for the elementary OH^- adsorption, namely, step (I) as the RDS.

Computational details of the hybrid solvent model

Solvents must be considered in the liquid-solid heterogeneous catalysis. Generally, the solvent effects can be treated using implicit (or continuum), cluster/continuum (or hybrid implicit/explicit), and fully explicit solvation models within DFT. Due to the lower computational cost, implicit solvent models, such as the Conductor-like Screening Model (COSMO), have been widely used for computational catalysis. However, implicit models can not directly model solvent molecules, while there are strong interactions between Na cations in PANa systems with the water molecules, and thus the implicit models are not a good choice. In our work, we used the hybrid (or cluster-continuum) approach which combines explicit and implicit solvation models and thus brings together key advantages of both explicit and implicit models.[3]

In the hybrid model, the solvation effect is treated by including (i) several explicit water molecules as the core solvation shell, and (ii) an implicit continuum solvation model to describe the long-range electrostatic interaction from water solution.

The explicit water solvation shell is generated using the Amorphous Cell Tools.[4, 5] The Amorphous Cell Tools build molecules in a cell in a Monte Carlo fashion by minimizing close contacts between atoms, whilst ensuring a realistic distribution of torsion angles for any given forcefield. For the unsupported PANa model, a large supercell was created. Then Amorphous Cell Tools were used to distribute water molecules inside the supercell with a density of 1 g/cm^3. The cell and outer shell water molecules were deleted afterwards to build the unsupported PANa model with explicit 16 water molecules. These 16 water molecules were finally preserved because previous studies demonstrated that the close water shell of Na cation contains four water molecules.[6, 7] Subsequently, the obtained structure was fully optimized at the DFT level of theory. Similar procedures were used to obtain the graphene supported PAN with explicit water solvation shell.

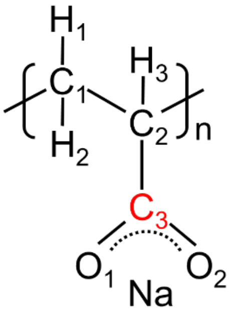

Site	Charge (e)
C_1	-0.057
C_2	-0.032
C_3	0.195
H_1	0.028
H_2	0.033
H_3	0.018
O_1	-0.226
O_2	-0.248

Supplementary Fig. 1: **Molecular structure and charge distribution of different atoms in PANa polymer chain**. The C_3 site possesses a positive charge of +0.195 e, thus serving as the possible active sites for OER; the highest density (1 out of every 3) of the positive carbon site makes PANa hydrogel an ideal material for OER.

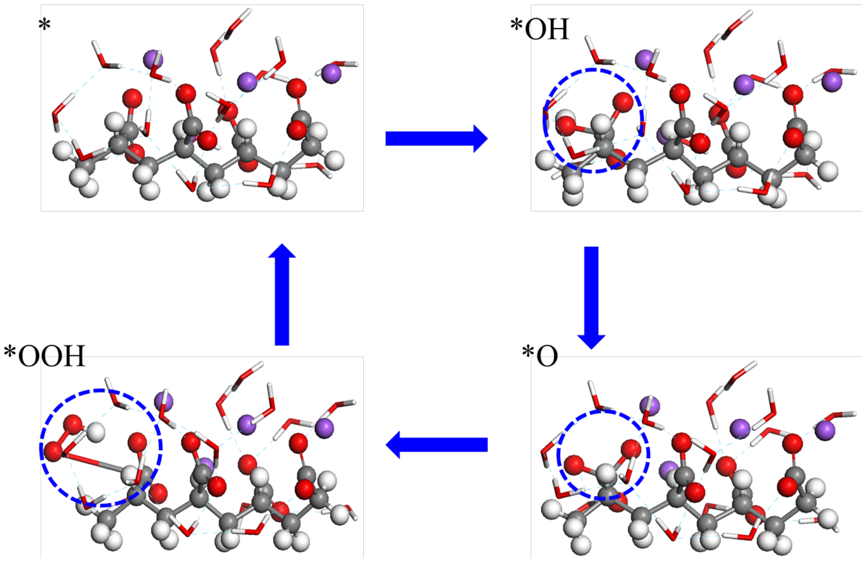

Supplementary Fig. 2: **Reaction pathway and binding configurations of *OH, *O, and *OOH on the left carboxyl carbons (blue dashed circles) of bare PANa**. Gray, red, purple, white represented carbon, oxygen, sodium, and hydrogen elements.

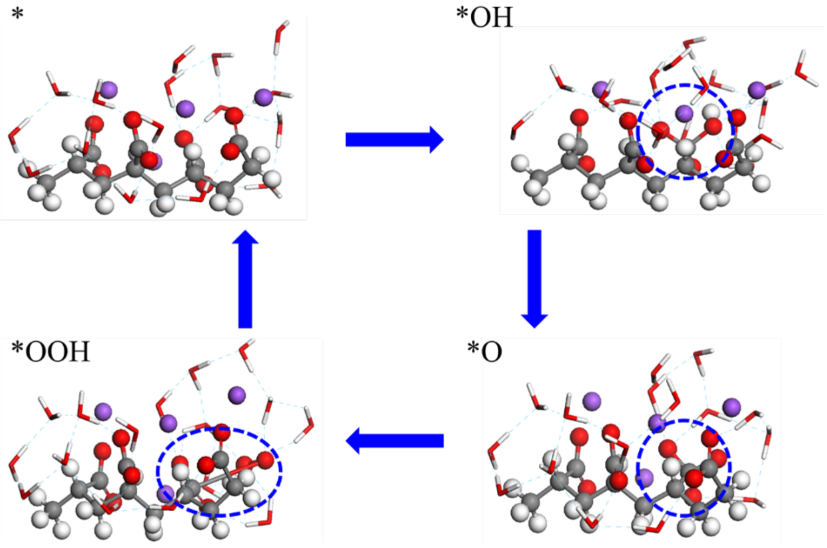

Supplementary Fig. 3: **Reaction pathway and binding configurations of *OH, *O, and *OOH on the middle carboxyl carbons (blue dashed circles) of bare PANa**. Gray, red, purple, white represented carbon, oxygen, sodium, and hydrogen elements.

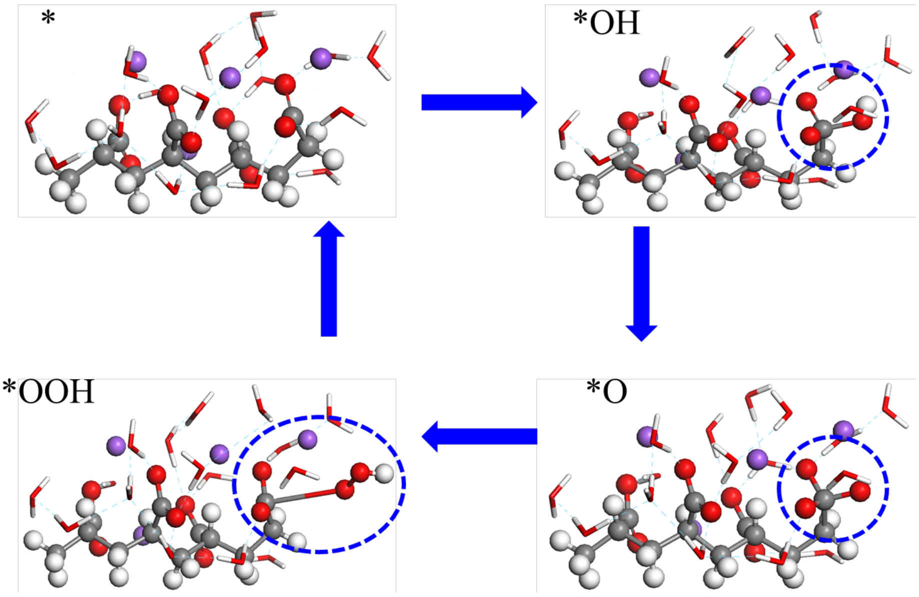

Supplementary Fig. 4: Reaction pathway and binding configurations of *OH, *O, and *OOH on the right carboxyl carbon (blue dashed circles) of bare PANa. Gray, red, purple, white represented carbon, oxygen, sodium, and hydrogen elements.

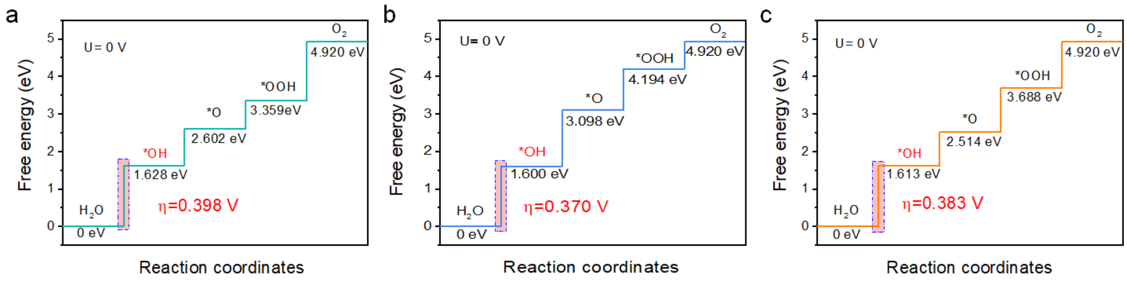

Supplementary Fig. 5: **Free energy diagrams**. **a** The left, **b** the middle, and **c** the right positive carboxyl carbon sites in PANa for OER. The optimised structures are shown in Figures S2-S4. The comparable results here suggest the positive carbon sites in PANa hydrogel can be deemed as equivalent.

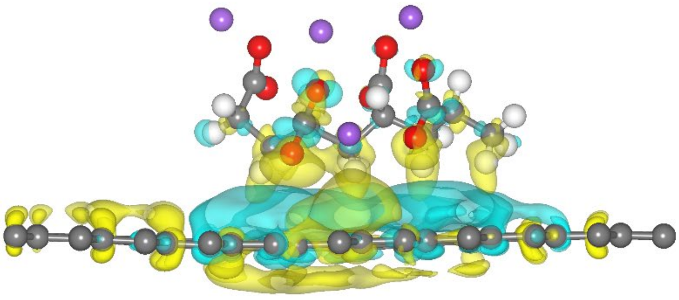

Supplementary Fig. 6: **Differential charge densities of the graphene supported PANa molecule**. Yellow and cyan isosurfaces represent for electron accumulation and depletion, respectively. Gray, red, purple, white represented carbon, oxygen, sodium, and hydrogen elements.

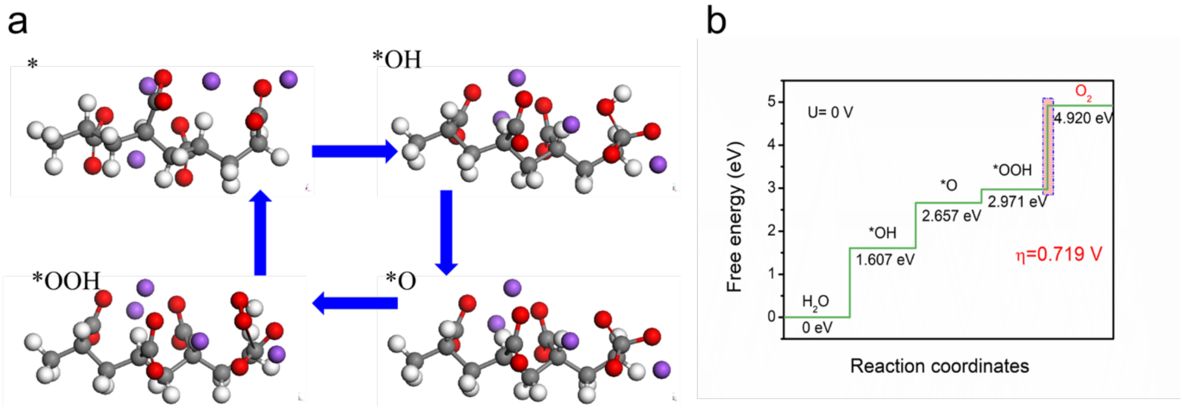

Supplementary Fig.7: **OER on the solvated water-free PANa model. a** Reaction pathway and binding configurations of *OH, *O, and *OOH on the carboxyl carbon of bare PANa without solvated water molecule. **b** Free energy diagrams. Gray, red, purple, and white represented carbon, oxygen, sodium, and hydrogen elements.

Note 1: For DFT calculations in this work we considered the water environment, because it has been demonstrated that the hydrogen bonding of the surrounding water plays significant roles in modulating the reaction intermediates, and previous oversimplified DFT calculations based on the vacuum environment probably cannot reveal the true activity of given electrocatalysts.[8, 9] Therefore, in Supplementary Fig. 2-5, water-solvated PANa models were adopted. For reference, we have also compared the simplified gas/solid interface data by excluding the presence of water. Supplementary Fig. 7 shows that for a typical carboxylate carbon site within the PANa backbone, an overpotential of 719 mV was required to drive OER, which is substantially larger than the water-free model equivalents (Supplementary Fig. 5). Such a substantial increase could be assigned to the excess binding energy of the *OOH species (Supplementary Fig. 7b). This result suggests the solvation water molecules can indeed interact with intermediates to minimize the overpotential, similar to the results in some recent works.[10, 11]

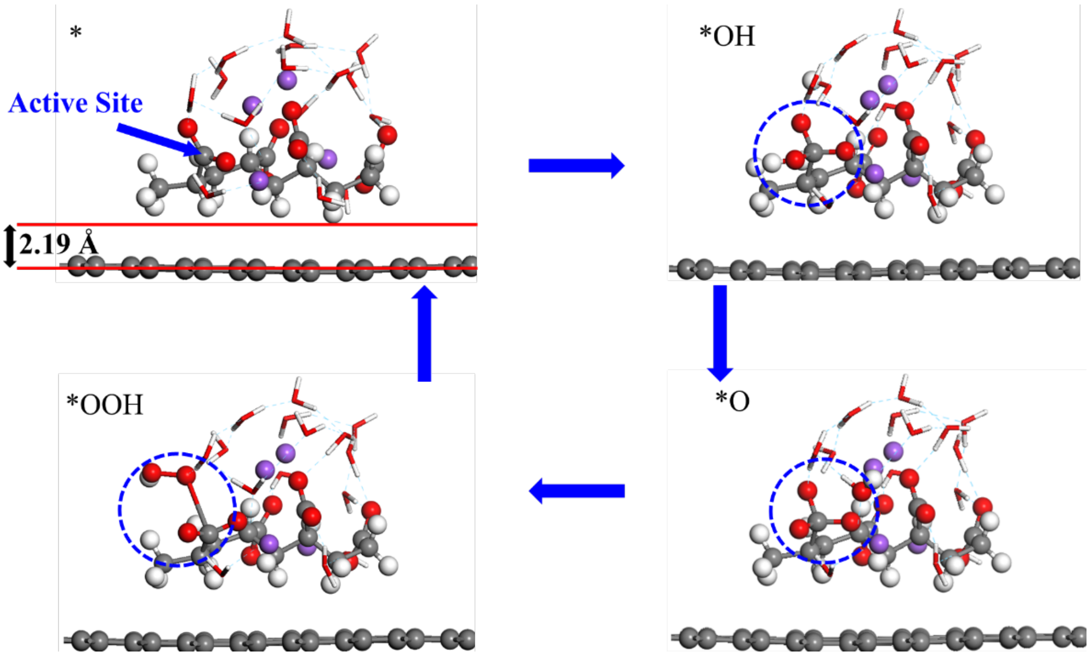

Supplementary Fig. 8: **Reaction pathway and binding configurations of *OH, *O, and *OOH on the carbons (blue dashed circles) of PANa with graphene substrate**. Gray, red, purple, white represented carbon, oxygen, sodium, and hydrogen elements.

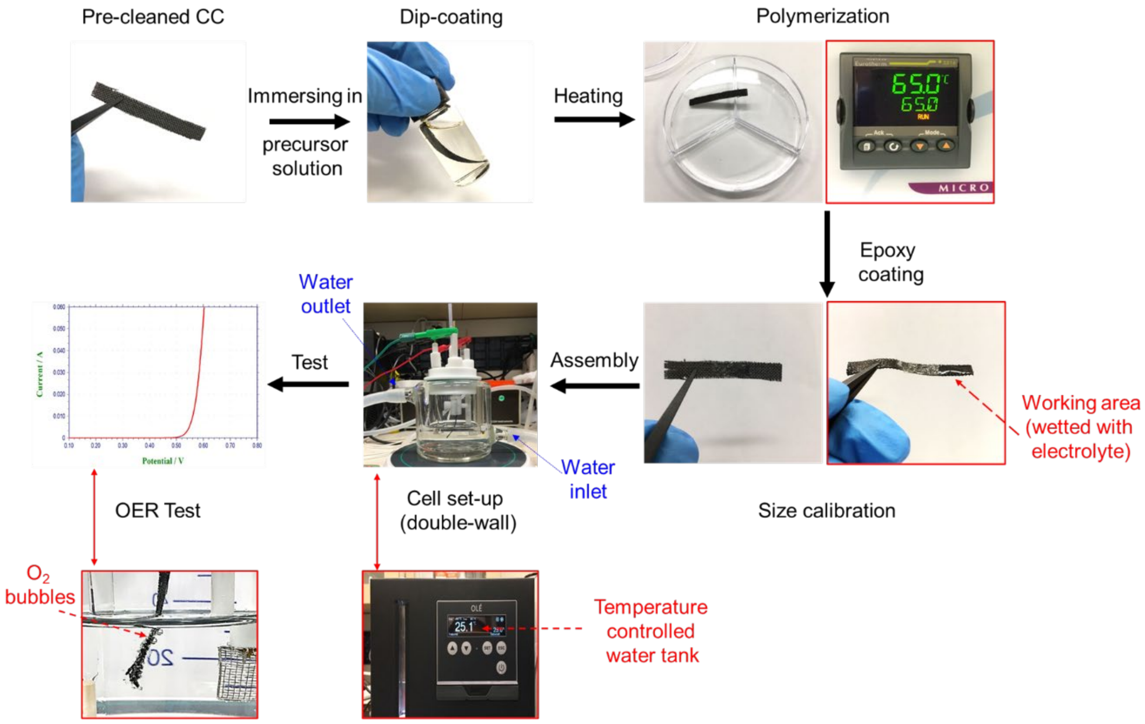

Supplementary Fig. 9: **Synthetic procedures and test set-up for the CC-PANa electrodes.**

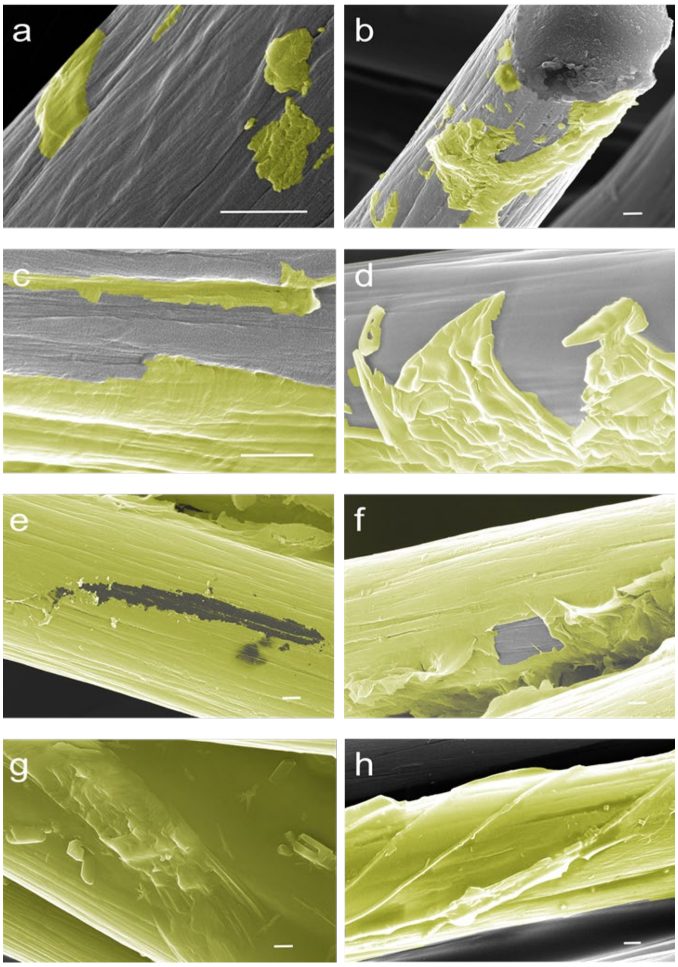

Supplementary Fig. 10: **SEM observation of the electrodes with different PANa coating amounts on CC substrate**. **a, b** CC-PANa-0.2. **c, d** CC-PANa-0.4. **e, f** CC-PANa. **g, h** CC-PANa-2. Scale bar: 1 μm in all figures.

Note 2: The content of the coated PANa hydrogel can be controlled by changing the initial concentration of AA precursor. For CC-PANa-0.2 electrode, the carbon fibers are partially patched with PANa hydrogel (Supplementary Fig. 10a-b), whilst more parts of the fibers in CC-PANa-0.4 electrode are covered (Supplementary Fig. 10c-d). The CC-PANa-2 electrode, on the contrary, is densely wrapped by excessive PANa (Supplementary Fig. 10g-h). As can be envisaged, the limited PANa hydrogel coverage will result in poor OER activity (since bare CC is almost relatively inactive), yet overwrapping of PANa can incur sluggish diffusion of the electrolyte and evolved gas. The CC-PANa electrode, with an optimal amount of PANa (Supplementary Fig. 10e-f), is expected to balance the concentration of active sites and diffusion kinetics.

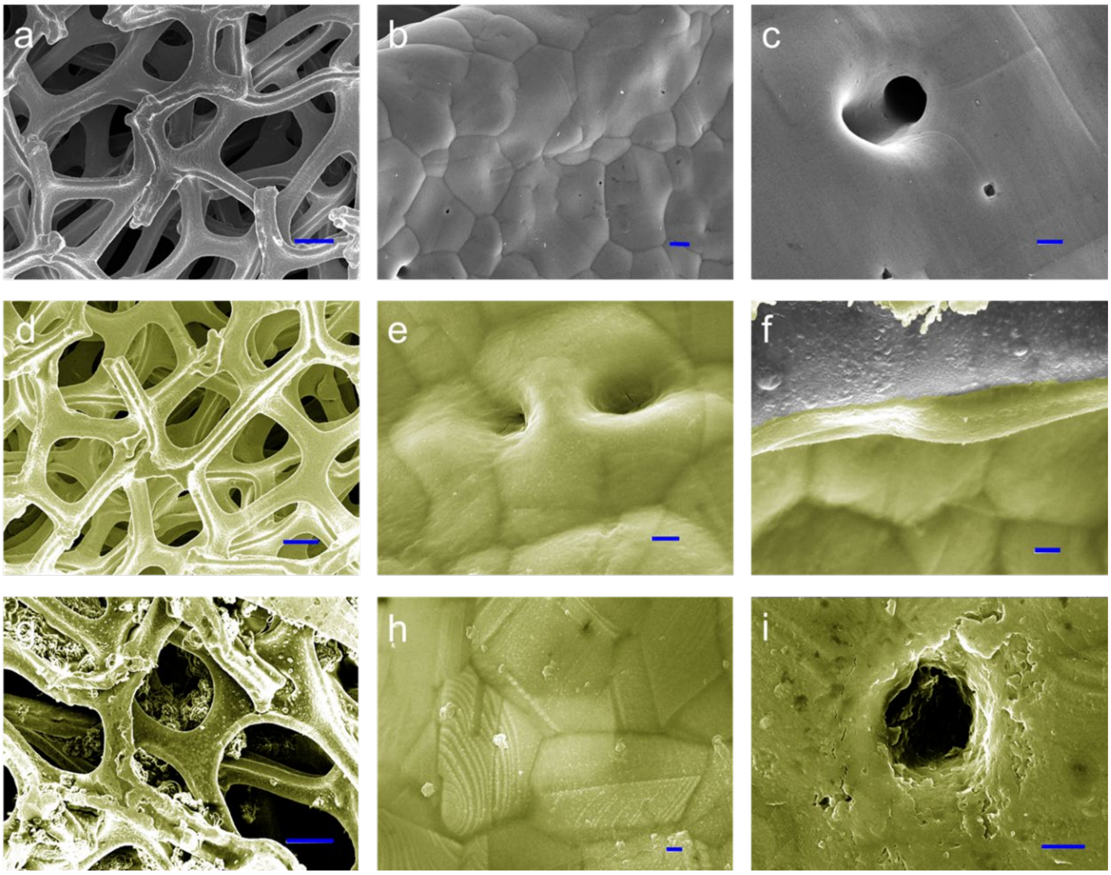

Supplementary Fig. 11: **SEM observation of the electrodes with different PANa coating amounts on CuF substrate. a-c** bare CuF. **d-f** CuF-PANa-0.4. **g-i** CuF-PANa. Scale bars: 100 μm in **a**, **d**, **g**; 1 μm in all others.

Note 3: Similar to the case of CC, the PANa hydrogel also has an optimal concentration when coating on CuF substrate. However, due to the much larger diameter of the Cu skeleton (thus the much smaller surface area), the CuF-PANa-0.4 electrode affords the best balance between the hydrogel coverage and thickness. As can be seen in Supplementary Fig. 8e-f, the PANa was uniformly masked onto the Cu skeleton, with a typical thickness of tens of nanometers.

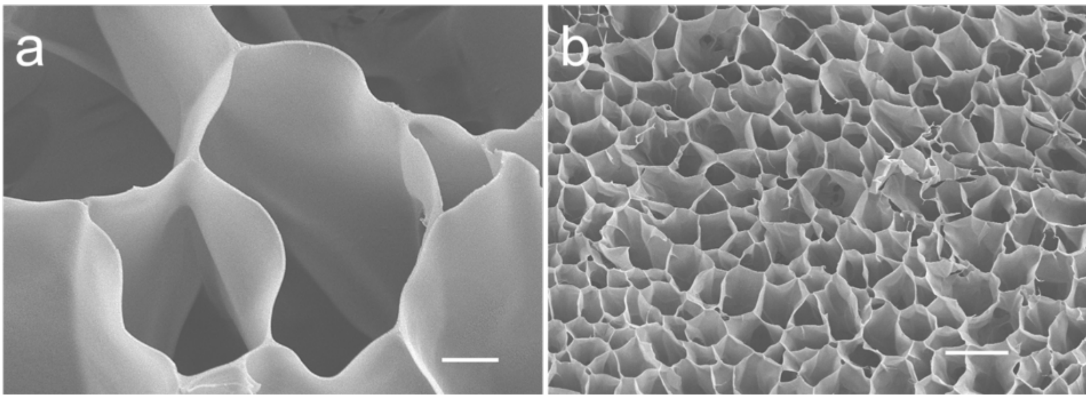

Supplementary Fig. 12: **Morphology of the freeze-dried PANa hydrogel at different magnifications**. Scale bars: **a** 500 nm, **b** 5 μm.

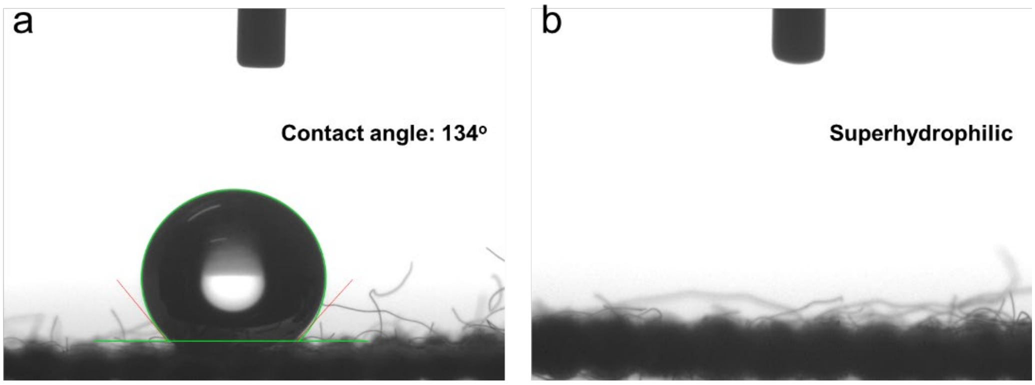

Supplementary Fig. 13: **Hydrophilicity tests**. Contact angles of a drop of water on **a** bare CC and **b** the CC-PANa electrodes.

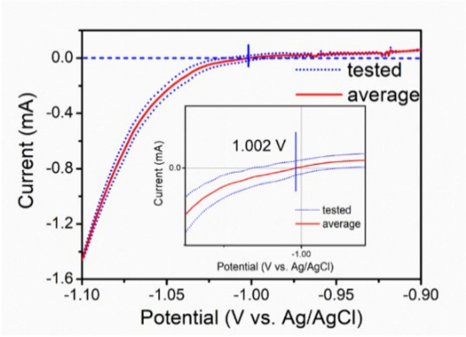

Supplementary Fig. 14: **Electrode calibration**. Calibration curves of the reference electrode (Ag/AgCl filled with saturated KCl solution) in H_2-saturated 1 M KOH solution (pH=13.6).

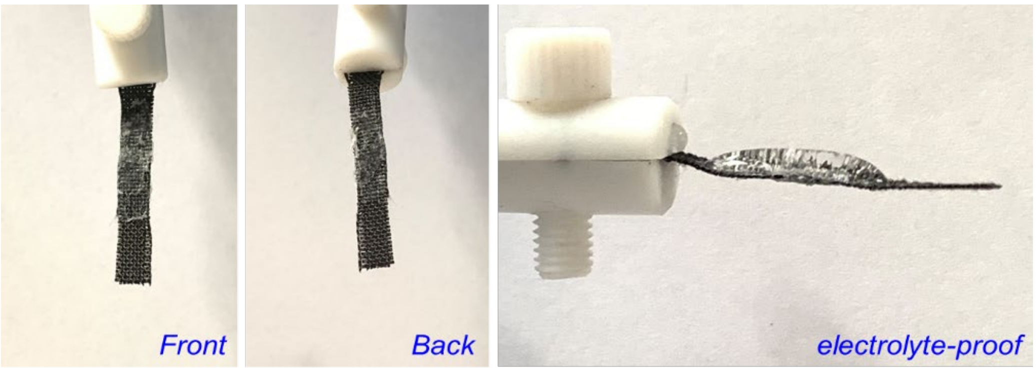

Supplementary Fig. 15: **The epoxy coated CC-PANa electrode showing electrolyte-proof effect in the coated region**.

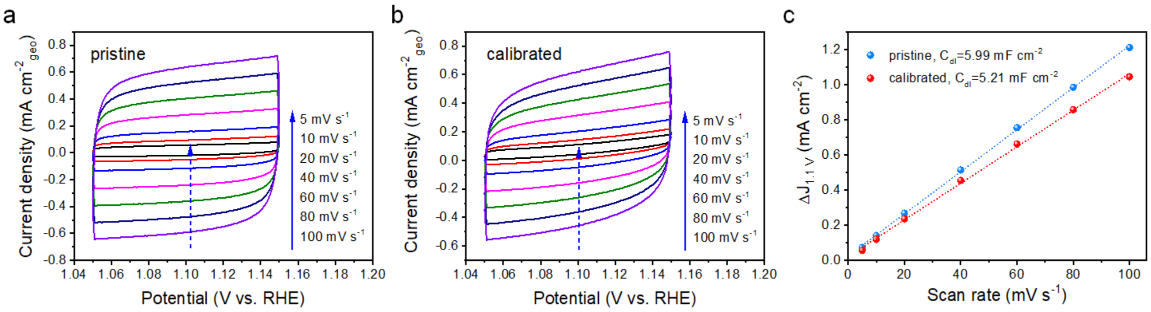

Supplementary Fig. 16: **Calibration of the working electrode by epoxy coating.** CV curves of **a** pristine and **b** calibrated (by epoxy-coating) CC-PANa electrodes recorded in a potential window between 1.05 and 1.15 V (vs. RHE) at the scan rates from 5 to 100 mV s^{-1}; (c) capacitive current density as a function of scan rates of different catalysts, with the double layer capacitance values listed.

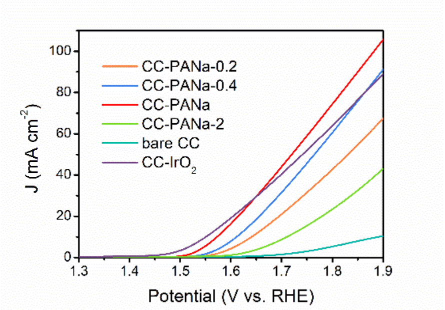

Supplementary Fig. 17: **Polarization curves of different electrodes in 1 M KOH without iR-compensation**.

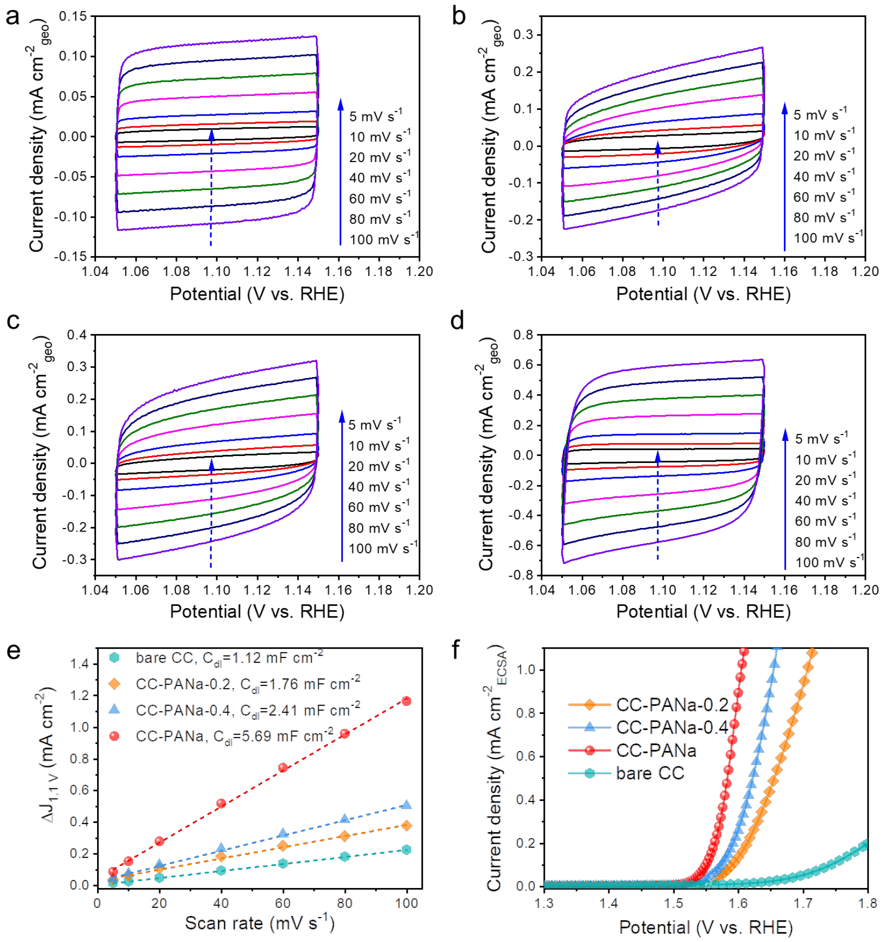

Supplementary Fig. 18: **OER current normalization by electrochemical surface area (ECSA).** CV curves of **a** bare CC, **b** CC-PANa-0.2, **c** CC-PANa-0.4, **d** CC-PANa electrodes recorded in a potential window between 1.05 and 1.15 V (vs. RHE) at the scan rates from 5 to 100 mV s^{-1}. **e** Capacitive current density as a function of scan rates of different catalysts, with the double layer capacitance values listed. **f** The normalized OER current density according to their ECSAs.

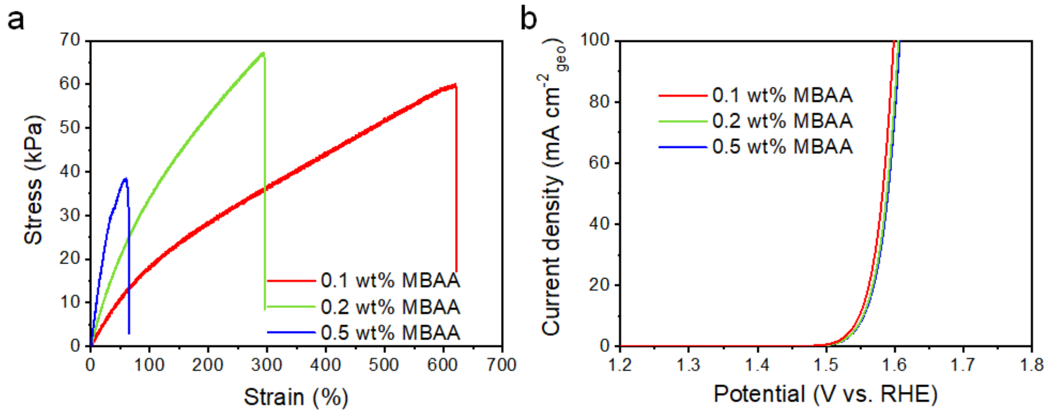

Supplementary Fig. 19: **Influence of the crosslinker concentration**. **a** Tensile strain-stress curves of the bare PANa hydrogel with different concentration of MBAA cross-linker. **b** OER polarization curves of the CC-PANa electrode synthesized with different concentration of MBAA cross-linker.

Note 4: The possible influence of the concentration of the crosslinker was compared. A series hydrogel-based electrodes were fabricated with different contents of the MBAA crosslinker: 0.1 wt%, 0.2 wt% and 0.5 wt%. As shown in Supplementary Fig. 19, the concentration of the crosslinker do affect the mechanical strength of the pure PANa hydrogel, but the OER activity of the CC-PANa electrodes is very similar. Hence, it can be deduced that the crosslinker, at least within the studied concentration range, plays a very minor role in determining the OER activity of the corresponding hydrogel.

The DFT calculations suggested that the carboxylate carbon atom is the only active site for OER. However, the family of acrylate-based hydrogels is huge. The reason why the PANa one was studied is that it has one of the highest densities (1 out of every 3) of positively charged carbon atoms, the theoretic active sites toward OER, in the polymer chain. To further review the generality of this conclusion, the OER performance of another two common acrylate-based hydrogels, sodium poly-methacrylate (PMANa) and poly-hydroxyethylmethacrylate (PHEMA), was compared. The structure of the two polymers, along with that of PANa, are shown in Supplementary Fig. 20a.

It is obvious that the density of the active site gradually decreases in the sequence of PANa (1/3), PMANa (1/4), and PHEMA (1/6). In the subsequent electrochemical tests (with an identical loading mass of the polymers), it was found that the OER performances also follow this sequence (Supplementary Fig. 20b, c). The apparent η_{10} was 316, 400, and 476 mV for PANa, PMANa, and PHEMA, respectively. Such a trend was consistent with their electrochemical impedance spectra and the normalized reaction current density (Supplementary Fig. 20d, e). These results suggest the acrylate-based hydrogels might be potential OER materials, and those with higher concentration of positive carbons can be more efficient in catalyzing OER.

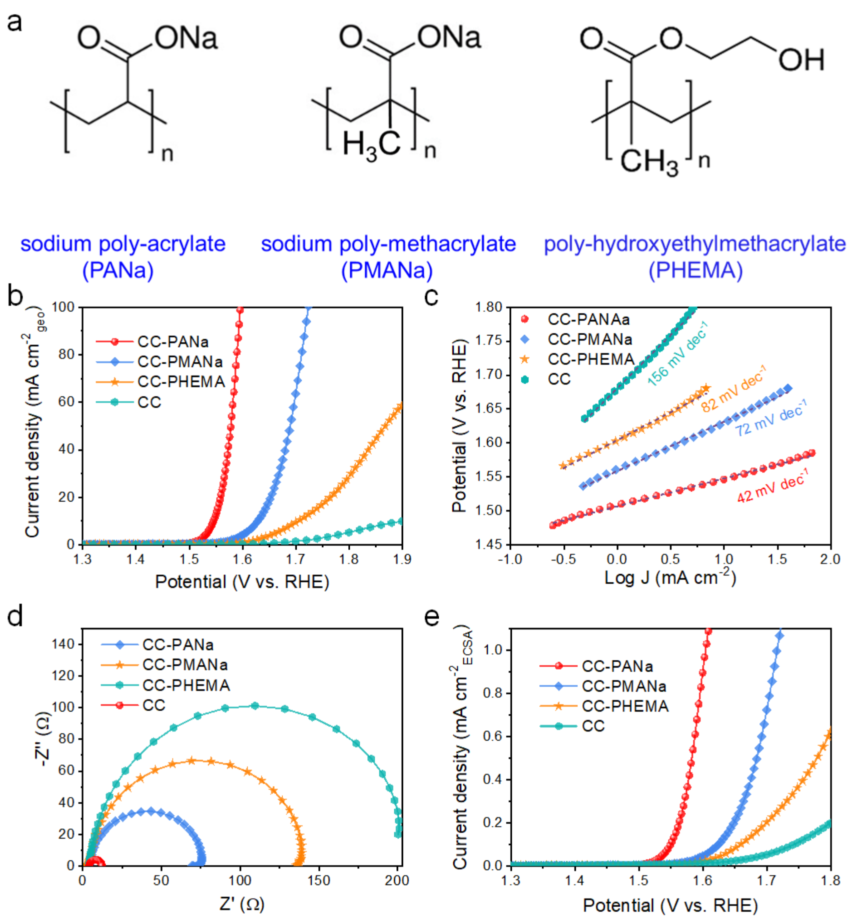

Supplementary Fig. 20: **Influence of the carboxylate group concentration. a** Molecular structure of three typical acrylate-based polymers. **b** Polarization curves and **c** corresponding Tafel slopes of different electrodes in 1M KOH. **d** EIS spectra. **e** Normalized (by ECSA) OER current density of the electrodes.

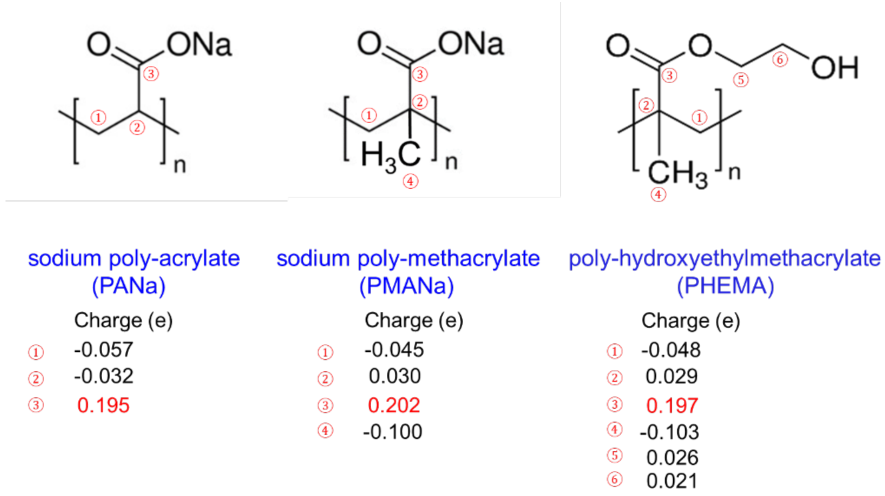

Supplementary Fig. 21: **Charge distribution of different carbon atoms in the three polymer chains.**

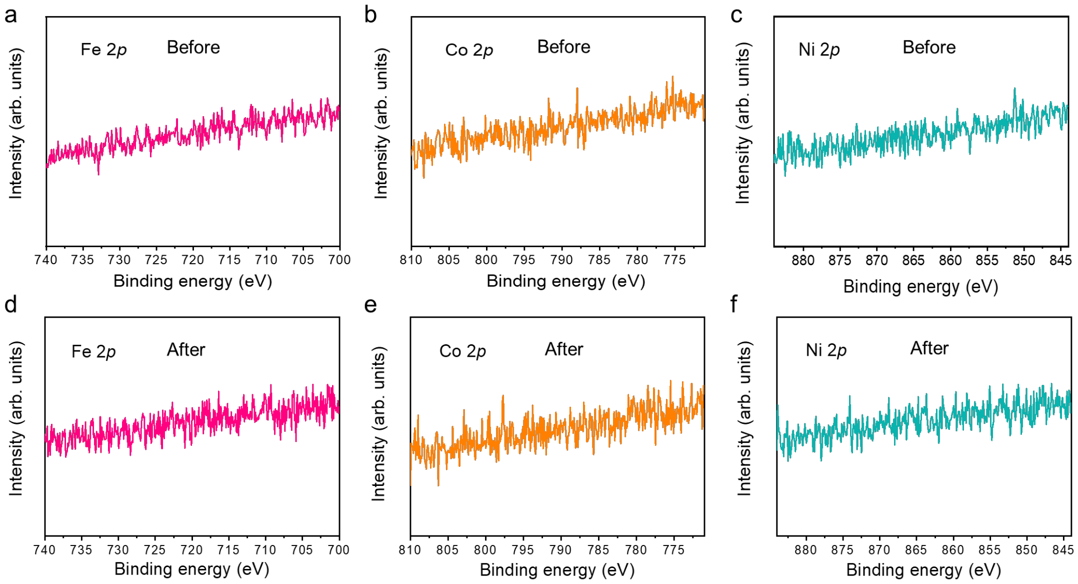

Supplementary Fig. 22: **XPS spectra of possible metal impurities (Fe, Co, Ni) within the CC-PANa electrode before and after OER tests**.

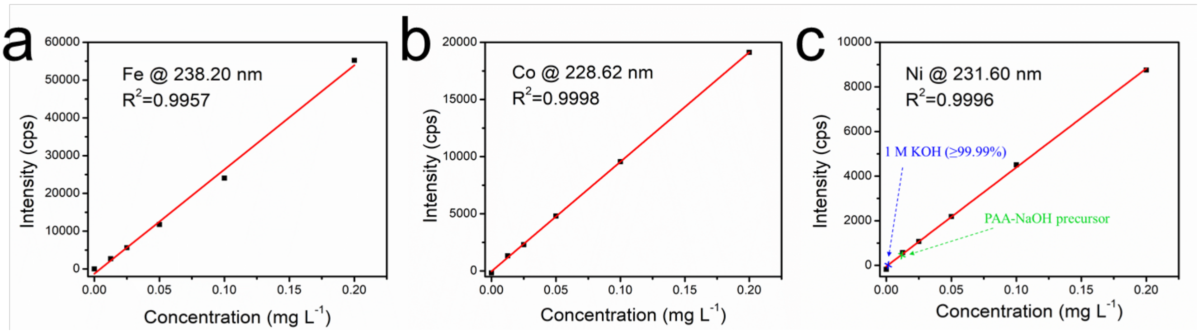

Supplementary Fig. 23: **Quantification of the possible metal impurities (Fe, Co, Ni) in the precursor and electrolyte by ICP-AES.** Calibration curves of **a** Fe, **b** Co, and **c** Ni elements in the precursor and electrolyte using ICP-AES. For Fe and Co elements, the signals showed a negative value, suggesting their concentration is below the detection limit (ppb) of the equipment. The detailed values of different metal ions are summarized in Supplementary Table 1.

Note 5: To determine the possible contamination by OER-active metal ions (e.g., Fe^{3+}, Co^{2+}, Ni^{2+}), we conducted a series of control experiments. XPS fine spectra in Supplementary Fig. 22 corroborate that no Fe, Co, or Ni species were detected in the CC-PANa sample before and after OER tests. Further, the inductively coupled plasma atomic emission spectroscopy (ICP-AES) element analysis reveals the concentrations of Fe and Co are below the detection limit (ppb) of the equipment (Supplementary Fig. 23, Supplementary Table 1). The Ni ion has an extremely small concentration of several μg L^{-1}, far below the smallest concentration (~ppm level) that could affect OER.[12, 13] Moreover, poisoning tests were conducted, but the results in Supplementary Fig. 24a exhibit that the OER activity of the CC-PANa electrode was not affected at all. These results validate that the contribution from the possible metal impurities is negligible.

It should also be noted that an ultrapure KOH (≥99.99%) was used for all OER tests to eliminate the possible contamination of the metal ions to the least level. In fact, we have additionally tested the OER activity of a used CC-PANa electrode in a normal KOH (≥90%)-based electrolyte to deliberately check the influence of the trace amount of metal ions (especially Ni^{3+}). However, as shown in Supplementary Fig. 24b, the OER activity of the electrode remained almost identical to that in the ultrapure KOH-based electrolyte, further suggesting that the contribution from the metal species toward OER can be excluded. Taken together, these control experiments verify that the PANa hydrogel (more specifically, the positive carbon within the carboxylate groups) is the intrinsic active site for OER.

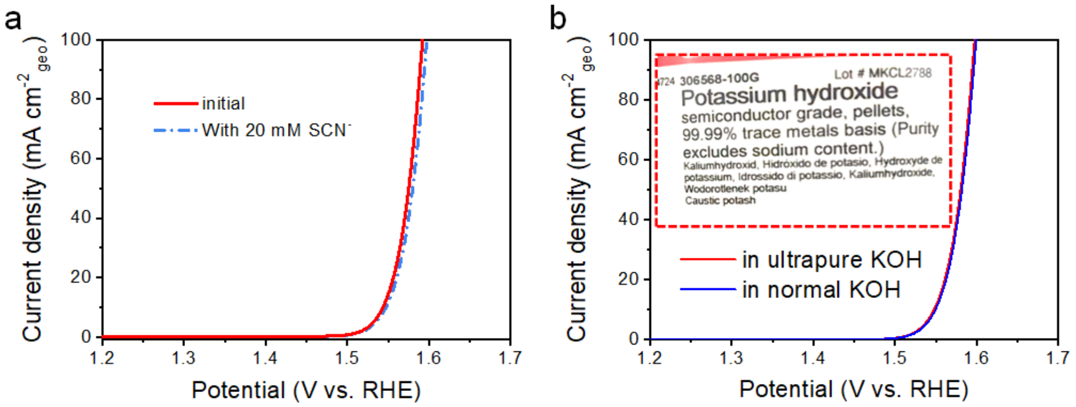

Supplementary Fig. 24: **Poisoning test and influence of the KOH purity**. **a** Polarization curves of the CC-PANa electrode with/without the presence of 20 mM KSCN. **b** Polarization curves of the electrode ultrapure KOH (≥99.99%, used in all other tests in this work) and in normal KOH (≥90%, only used here for comparison) electrolytes, the inset shows the content information of the ultrapure KOH (provided by the manufacturer, Sigma-Aldrich).

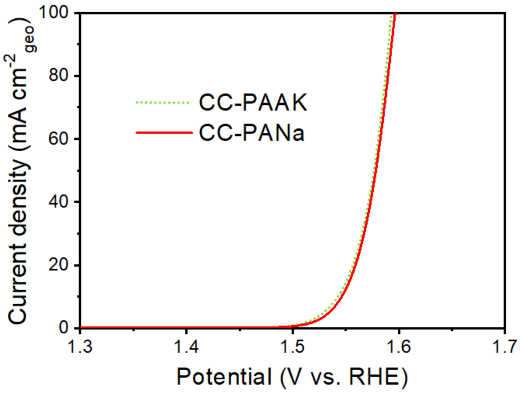

Supplementary Fig. 25: **OER performance of the CC-PANa and CC-PAK electrodes**. The almost identical OER activity indicates that the replacing of Na^+ with K^+ has no effect on the electrocatalytic activity of the polymeric hydrogel backbone.

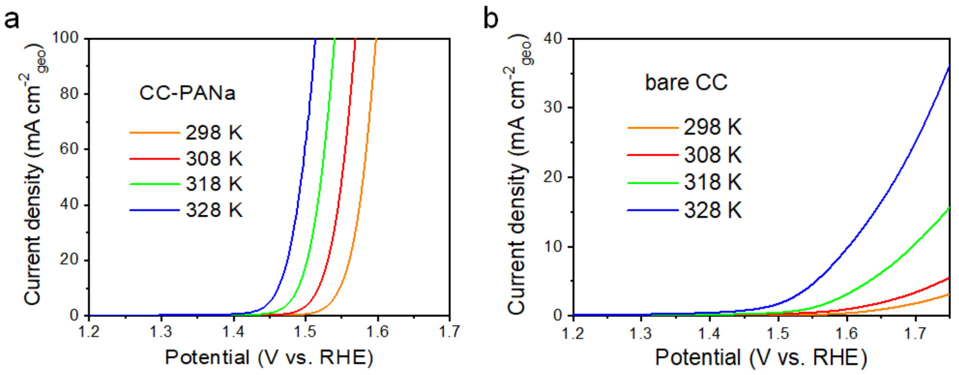

Supplementary Fig. 26: **Temperature-dependent polarization curves** of **a** CC-PANa and **b** bare CC electrodes.

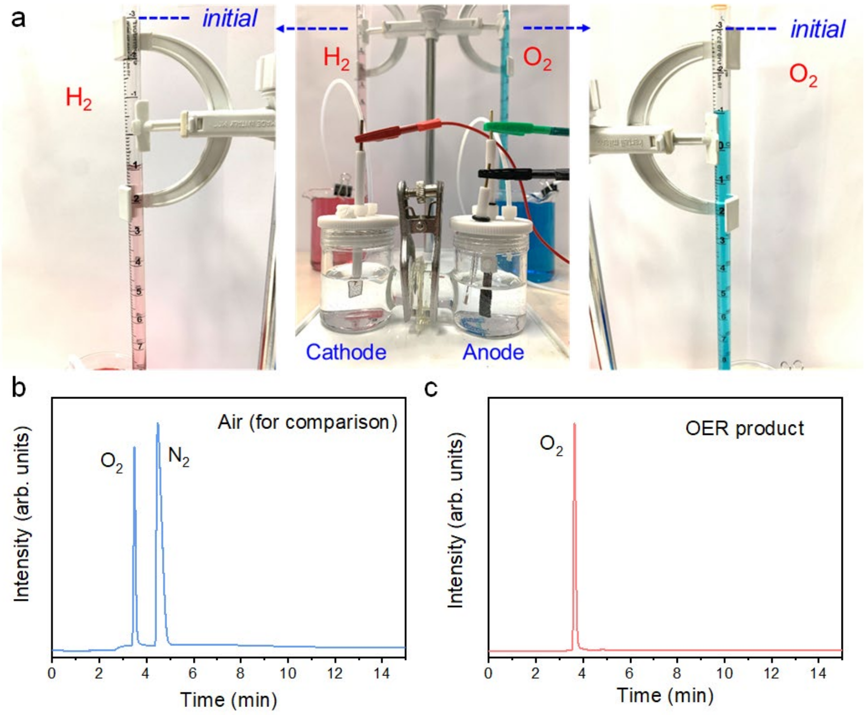

Supplementary Fig. 27: **Quantification of Faradaic efficiency of the CC-PANa electrode toward OER. a** Cell set-up for determining the Faradaic efficiency of OER using the volumetric method. **b** GC signal recorded from air. **c** GC signal recorded from the gaseous OER product in the top of the graduated tube.

Note 6: A volumetric method deploying a graduated tube was adopted to measure the evolved gas (method described in the experimental section) and determine the Faradaic efficiency (FE) of OER. A typical snapshot of the volume of generated H_2 and O_2 was shown in Supplementary Fig. 27a. The FE was determined to be 98.5% (Figure 3f). After the course of electrolysis, we detected the evolved gas from the top of the anode side using a gas chromatogram (GC, Shimadzu GC2030). With a detection limit of ca. 10 ppm, the GC showed the oxygen was the only product without any impurities (e.g., CO, the possible oxidation product of carbon). Therefore, we can safely conclude that the anodic current of the PANa-based electrode was from the electrocatalytic OER process.

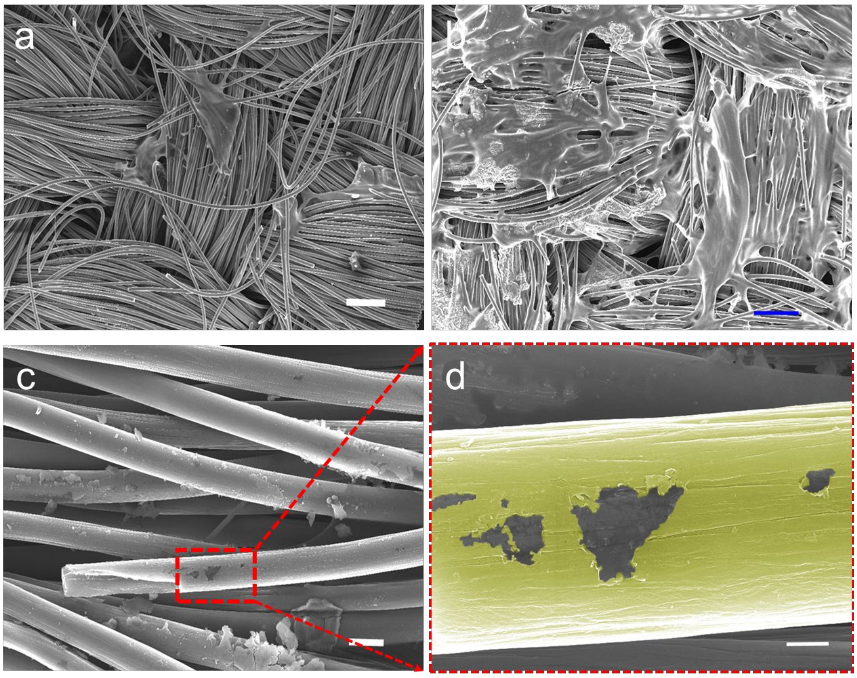

Supplementary Fig. 28: **Morphology of the electrode after OER**. SEM images with different magnifications of the CC-PANa electrode after OER test. Scale bar: **a** and **b** 100 μm; **c** 10 μm; **d** 2 μm.

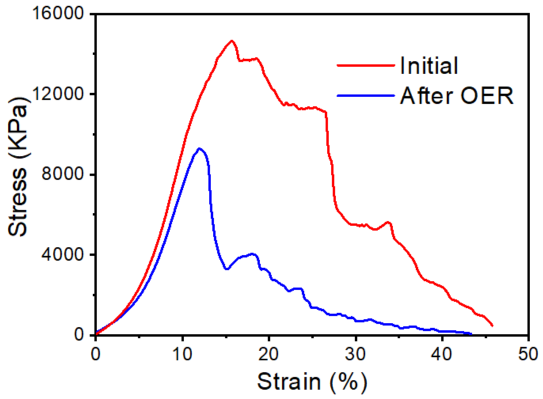

Supplementary Fig. 29: **Tensile stress-strain curves of the CC-PANa electrode before and after OER test**.

Note 7: The typical tensile strain-stress test of the CC-PANa electrode was performed before and after OER test. As shown in Supplementary Fig. 29, the pre- and post-test (48 h) electrode showed a maximum stress of 14,690 and 9,286 KPa, respectively, together with a similar strain of ca. 45%. The decreased stress was reasonably caused by the inevitable swelling of the PANa gel, which then slightly detached from the carbon fibers and also distorted the aligned carbon fibers of the CC substrate. As such, SEM was used to observe the post-OER electrode. The PANa gel indeed swelled and showed more irregular aggregates after OER, but most of the fibers were still well encapsulated by PANa (Supplementary Fig. 28).

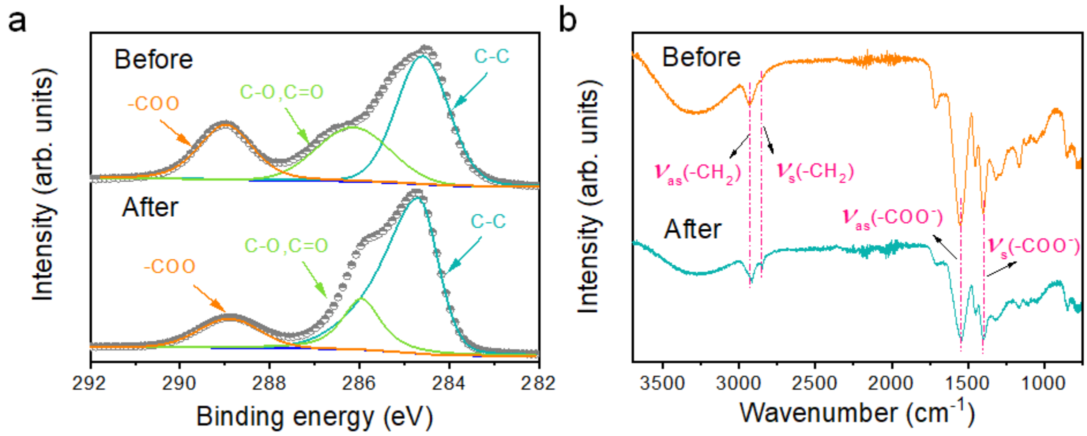

Supplementary Fig. 30: Chemical structure of the electrode before and after OER. **a** XPS C1*s* fine spectra and **b** ATR-IR spectra of the CC-PANa electrode before and after OER test.

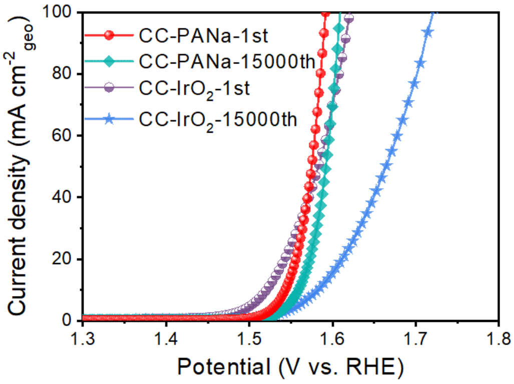

Supplementary Fig. 31: Polarization curves of the CC-PANa and CC-IrO$_2$ electrodes before and after 15000 cyclic scans.

Note 8: The OER stability test of CC-IrO$_2$ electrode was evaluated as a reference. As shown in Supplementary Fig. 31, after 15000 cyclic scans (1.2-1.7 V vs. RHE, totalling 41.66 h), the CC-IrO$_2$ electrode displayed obvious performance decay (η_{10} and η_{100} increased by 65 and 108 mV, respectively.) By contrast, the CC-PANa electrode was much more stable (η_{10} and η_{100} increased by 16 and 17 mV, respectively). The enhanced stability of the CC-PANa electrode can be reasonably credited to the relatively intimate coupling of the hydrogel film onto the CC substrate, which avoids the possible dissolution/detachment and ripening of the drop-casted IrO$_2$ nanoparticles during OER.

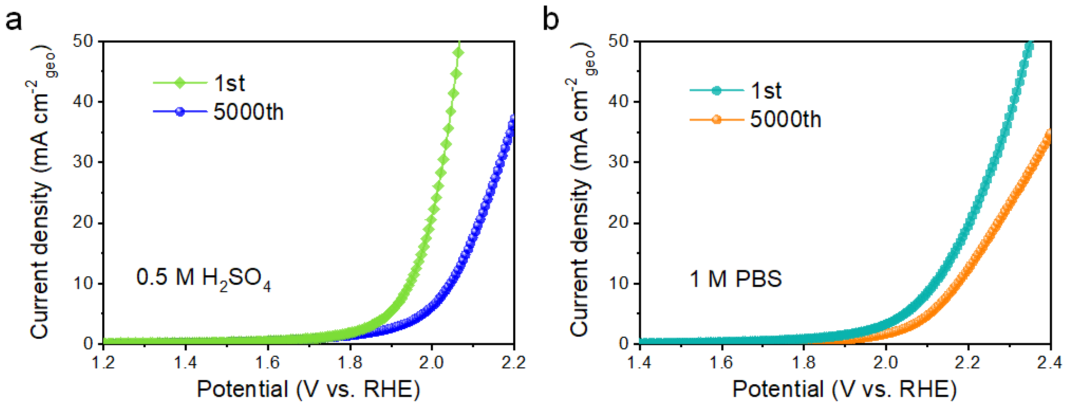

Supplementary Fig. 32: **Stability of the electrode in different electrolytes**. Polarization curves of the CC-PANa electrode before and after cyclic scans in **a** 0.5 M H_2SO_4 and **b** 1 M PBS electrolytes.

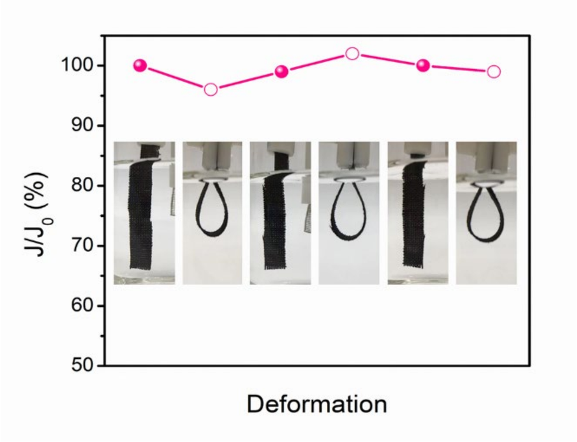

Supplementary Fig. 33: **The electrode's OER activity upon deformations.** The variation in the OER current densities of CC-PANa electrode at 1.55 V (vs. RHE) in 1 M KOH during bending and release processes; insets show the electrode during the bending and release processes.

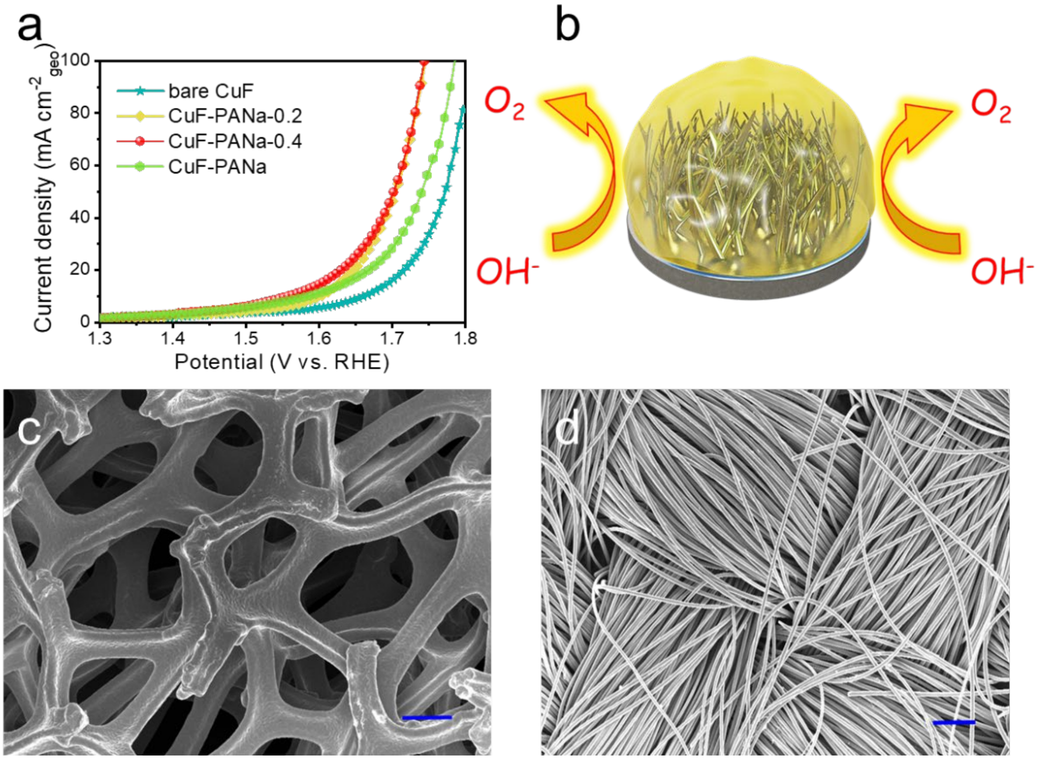

Supplementary Fig. 34: **OER activity of the CuF-PANa electrodes**. **a** Polarization curves of different content of PANa hydrogel-bearing CuF electrodes. **b** Schematic showing the advantage of CuF's hierarchical structure toward OER; typical SEM images showing the different effective surface areas of **c** CuF and **d** CC substrates. Scale bar: 100 μm in **c** and **d**.

Note 9: As expected, the CuF-PANa electrodes also exhibited obviously enhanced OER activity compared with bare CuF (Supplementary Fig. 34a). The CuF-PANa-0.4 electrode affords the best OER activity, with an η_{10} of 330 mV, meriting by its appropriate amount of coated PANa (Supplementary Fig. 11). The slight difference can be understood by the different diameters of the conductive substrates. As discussed in the manuscript, the PANa is exactly insulative, hence a conductive substrate is indispensable to active the hydrogel. The diameter of the Cu branches is much larger than that of the fibers in CC (Supplementary Fig. 34c-d), therefore the effective activated area within the PANa hydrogel is reduced. As a result, a "compatible" amount is required for CuF to deliver the optimal OER performance.

To further attest the intrinsic activity of the hydrogel for direct OER, we cast the hydrogel onto planar GCE. As shown in Supplementary Fig. 35, compared with bare GCE, the PANa-bearing GCE affords a remarkably larger reaction current. When a more conductive AuE was adopted, the OER current was further enhanced, similar to the case in OER-active metal compounds.[14] Though the compatible thickness was not optimized on the two planar electrodes and the normalized current density is comparatively small, the results here undoubtedly highlight the unexpected OER activity of the hydrogel materials.

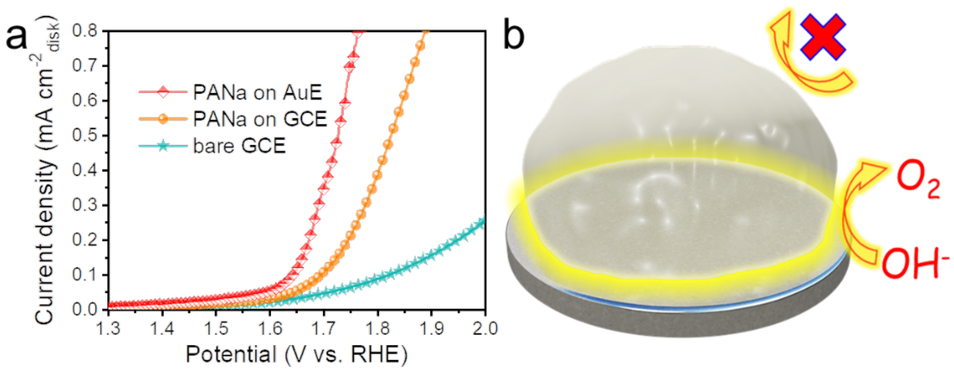

Supplementary Fig. 35: **The activity of PANa on planar electrodes**. **a** OER activity of PANa hydrogel on planar Au electrode (AuE) and glassy carbon electrode (GCE), with the bare GCE as a reference. **b** Schematic showing the limited active regions (highlighted in yellow) on planar electrodes. Note the thickness of the PANa hydrogel was not optimized on the AuE and GCE.

Note 10: Since the hydrogel is intrinsically insulative, hence a conductive substrate is indispensable for charge transfer. For the conductive substrates (CC, CuF, AuE and GCE) used in this work, all of them can afford satisfactory conductivity to deliver decent OER activity, despite metal substrates have higher conductivity than carbon ones. It was observed that the planar Au disk electrode loaded PANa gel showed slightly better OER activity (Supplementary Fig. 35a) compared with the GCE, similar to previous results.[3] However, as can be understood from Supplementary Fig. 35b, on the planar electrodes, the reactive sites are exclusively restricted to the edges. Hence, the PANa-coated AuE and GCE delivered much smaller reaction current densities (Supplementary Fig. 35a) compared with the CC-PANa one (Figure 3a). Despite so, the OER distinction on AuE and GCE indeed highlight that the substrate with a better conductivity can facilitate OER.

To compare the intrinsic activity of the electrode, it's more reasonable to use the CC substrate where most of the PANa hydrogel can actually contribute to OER. For the optimal CC-PANa electrode, the PANa ($[-CH_2-CH(CO_2Na)-]_n$) catalyst loading was 0.8±0.2 mg cm^{-2} (based on geometric area of the CC substrate, determined by a semi-micro balance) from batch to batch. Assuming an average loading of 0.8 mg cm^{-2}, the concentration of the sodium acrylate monomer is 8.51×10^{-6} mol cm^{-2}. At a typical overpotential of 350 mV, the reaction current density was 54.1 mA cm^{-2}, hence the turnover frequency (TOF) can be calculated as:[15, 16]

$$\text{TOF}=\frac{J\times A}{4\times F\times m}=\frac{54.1\times10^{-3}\times1}{4\times96485\times8.51\times10^{-6}}=1.65\times10^{-2}\ \text{s}^{-1}$$

where J is the reaction current density (A cm^{-2}), A is the electrode area (cm^{-2}), m is the number of moles of the active materials (all PANa molecules are assumed to participate in OER), and F is the faraday constant (96485 C mol^{-1}). Similarly, at 10 mA cm^{-2}, the TOF can be calculated as 3.04×10^{-3} s^{-1}.

Note these values are calculated on assumption that all coated PANa hydrogel participated in OER. However, due to its insulative nature, it is difficult figure out how many sites are actually involved, but it is obvious that only those in the vicinity of carbon substrate have actual contribution toward OER. In other words, these TOF values are somewhat underestimated.

For the CC-IrO_2 electrode, a typical 0.6 mg cm^{-2} of IrO_2 was loaded, equalling to a concentration of $0.6\times10^{-3}/224.22=2.68\times10^{-6}$ mol cm^{-2}. It is known that the IrO_2 material generally possesses a much higher electrical conductivity (10^{-2}-10^{2} S cm^{-1}),[17, 18, 19] and the particulate IrO_2 has a much larger accessible active surface area. Hence, it is fair to compare the TOF of CC-PANa and CC-PANa. At a

typical overpotential of 350 mV, the $CC\text{-}IrO_2$ delivered an oxygen evolution current density of 53.6 mA cm^{-2}, therefore the TOF can be calculated as:

$$TOF=\frac{J \times A}{4 \times F \times m}=\frac{53.6\times10^{-3}\times1}{4\times96485\times2.68\times10^{-6}}=5.18\times10^{-2}\ s^{-1}$$

It's evident that this value is comparable to (within the same order of magnitude) that of the CC-PANa electrode ($1.65\times10^{-2}\ s^{-1}$ at η=350 mV). Therefore, we can safely conclude that the hydrogel has an intrinsic OER activity on par with IrO_2.

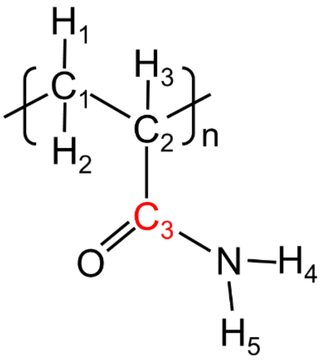

Site	Charge (e)
C_1	-0.064
C_2	-0.049
C_3	0.152
H_1	0.029
H_2	0.031
H_3	0.032
H_4	0.132
H_5	0.120
N	-0.151
O	-0.294

Supplementary Fig. 36: **Molecular structure and charge distribution of different atoms in PAM polymer chain.**

Note 11: The charge density of the carbon sites in PANa and PAM hydrogels was compared. The carboxylate carbon in PANa has a positive charge of 0.195e, higher than amide carbon of 0.152e (Supplementary Fig. 1, 36). It is well established that the positively charged carbon atoms can accept electrons from OH^- to form $^*OH$, which is the elementary step of OER.[20, 21] Therefore, a carbon site with higher positive charge density can potentially be beneficial for OER. This explains why the PANa has better OER activity than PAM based electrode, and why the PAM shows better OER activity upon hydrolyzation (because of more carboxylate groups in HPAM) (Figure 5).

Similarly, Supplementary Fig. 21 shows the carboxylate carbon sites have a charge density of 0.202e and 0.197e in PMANa and PHEMA, respectively, both very close to that of PANa. Therefore, all the carboxylate carbon sites are active toward OER, as shown in Supplementary Fig. 20. However, note the loading mass of all the hydrogels was controlled to be close (0.8 ± 0.2 mg cm^{-2}) for a fair specific activity comparison. In this context, the PANa hydrogel with the highest density of active site (1 out of every 3 carbon atoms) delivered better OER activity than the other two control hydrogel electrodes (1/4 and 1/6 active carbon for PMANa and PHEMA).

It should be noted that DFT calculation serves as a pre-screen descriptor to identify the possible active sites for OER: those with higher positive charges can be potential active sites. Therefore, the carboxylate carbon, with its most positive charge density, is the only possible site to exert OER (others can hardly adsorb the OH^-). Given the experimental results, it can be inferred that the possible threshold for the magnitude of positive charge leading to OER activity may be 0.20 e. This value is comparable to those in previously reported heteroatom-doped carbon electrocatalysts where the positive carbons are deemed as active sites.[22, 23]

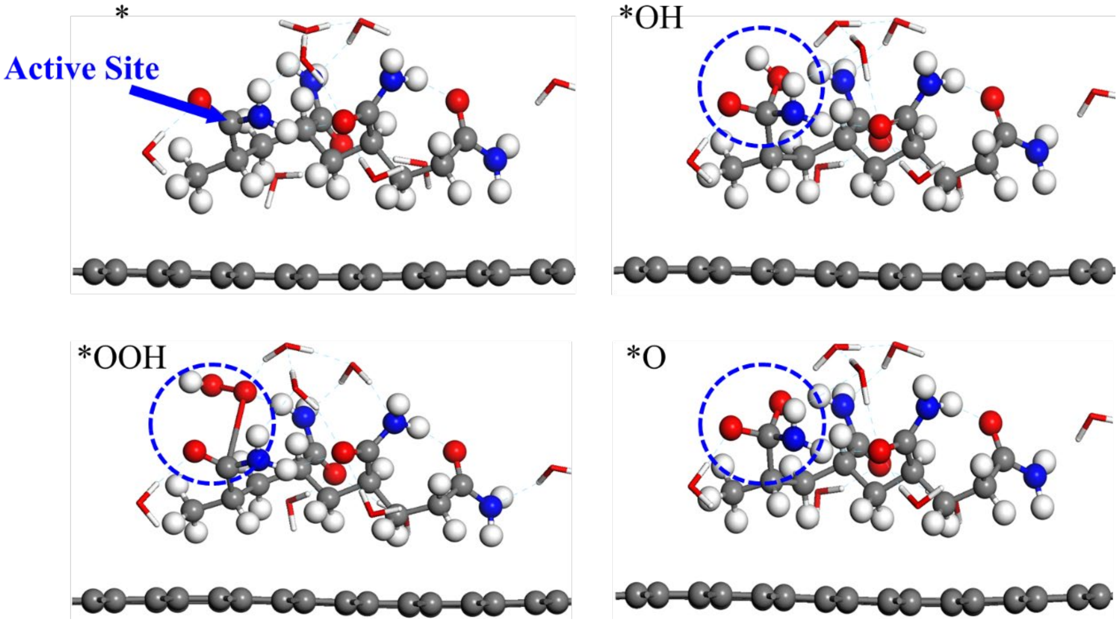

Supplementary Fig. 37: **Binding configurations of *OH, *O, and *OOH on the carbons (blue dashed circles) of PAM with graphene substrate**. Gray, red, blue, white represented carbon, oxygen, nitrogen, and hydrogen elements.

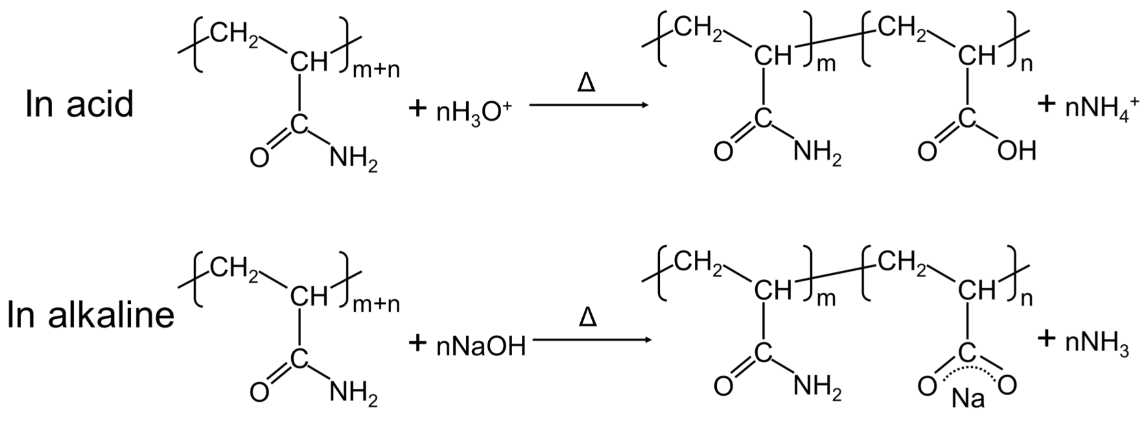

Supplementary Fig. 38: **Hydrolysis mechanisms of PAM under acidic and alkaline conditions.**

Note 12: Several essential control experiments were performed to justify the originality of the OER activity from the PANa hydrogel. First, before fabricating the CC-PANa electrodes, the CC substrate was calcinated at 300 °C for 1 h in air and washed with 6 M HCl followed by repeated thorough washing with DI water. The calcination followed by acid and water washing can effectively remove the metal impurities from the surface of CC.[24] Note HCl instead of oxidative HNO_3 was used to avoid introducing too much polar groups onto the carbon fiber surface. This pre-treatment could exclude the contribution of possible metal impurities but ensure the CC surface is relatively inert. XPS results in Fig. 2e confirmed that the pristine CC surface has a low content of polar groups like carbonyl and carboxylate. Fig. 3a-d show that the bare CC indeed delivered low OER activity (η_{10}= 640 mV, Tafel slope of 165 mV dec^{-1}). By contrast, after coating with PANa hydrogel, the CC-PANa electrode delivered substantially boosted OER activity. The CC therefore served as a reference sample with very limited activity.

Second, we have also coated the hydrogel onto copper foam, which is an OER-less active substrate (compared with Ni foam and stainless-steel foam). As shown in Supplementary Fig. 34, the PANa-bearing Cu foam delivered obviously enhanced OER activity comparable to that of the CC-PANa electrode. More significantly, for all the XANES tests, we did not use the CC substrate; the PANa hydrogel was coated onto the Cu foam to avoid signal contamination from CC (as carbon absorption spectra were recorded). However, we still observed the charge transfer between C and O in $-COO^-$ group, which is correlated with the OER process (Fig. 4b). Similarly, in the in-operando Raman tests, the hydrogel was coated onto a roughened Au substrate to detect the characteristic vibration of the superoxide intermediate (*O-O).[25] The fingerprint information of the superoxide of OER was observed, which was additionally corroborated by the subsequent isotope labelling test (Fig. 4d-e). Given that the influences of possible OER-active metal impurities were excluded (Supplementary Fig. 22-24), all these results therefore reveal that the PANa hydrogel is intrinsically active toward OER.

Third, another indirect evidence comes from the control experiments using another hydrogel, polyacrylamide (PAM), based electrodes (Fig. 5). If we assume the OER activity is mainly provided by CC, the anodic current from the PAM and PANa based electrodes should be identical (or at least very comparable) given their very similar chemical structures and loading masses. However, as shown in Fig. 5c-d, the anodic current of the two hydrogels-based electrodes was strikingly different. Besides, when subjected to a hydrolyzing treatment to introduce more carboxylate groups, the hydrolyzed PAM electrode (CC-HPAM) delivered much improved OER activity compared with pristine CC-PAM (Fig. 5c-d). These results obviously contradict the assumption that CC is the active sites, and therefore

suggesting that the observed OER current is rendered by the PANa hydrogel moiety (more specifically, the carboxylate carbon sites).

Supplementary Table 1. Detected concentrations of OER-active metal impurities by ICP-AES

	Fe (mg L^{-1})	Co (mg L^{-1})	Ni (mg L^{-1})
1 M KOH(≥99.99%)	-0.0138*	-0.0034*	0.0024
PAA-KOH (≥99.99%) precursor	-0.0284*	-0.0032*	-0.0068*
PAA-NaOH precursor	-0.0254*	-0.0012*	0.0133

*: A negative value suggests the concentration of the metal impurity is below the detection limit (ppb) of ICP-AES.

Supplementary Table 2. Comparison study of some metal-free electrocatalysts for OER in alkaline electrolytes

Catalyst	Form	η_{10} (mV)	Tafel slope (mV dec^{-1})	electrolyte	Reference
CC-PANa	Free-standing	316	42	1 M KOH	This work
N,O-doped carbon film	Free-standing	360	141	0.1 M KOH	Adv. Mater. 2014, 26, 2925
P-C_3N_4/CC	Free-standing	400	62	0.1 M KOH	Angew. Chem. Int. Ed. 2015, 54, 4646
N, S-doped graphite foil	Free-standing	346	78	1 M KOH	Adv. Energy Mater. 2016, 6, 1501492
PEMAc@CNTs90	Free-standing	300	52	1 M KOH	Energy Environ. Sci. 2017, 10, 2312
CC-PPPI	Free-standing	470	75	0.1 M KOH	Angew. Chem. Int. Ed. 2018, 57, 12563
PA-PPy/CC	Free-standing	340	55	1 M KOH	Angew. Chem. Int. Ed. 2019, 58, 4318
Activated CC	Free-standing	360	52	1 M KOH	Adv. Energy Mater. 2019, 9, 1802936
g-C_3N_4 NS–CNT	Powder	370	83	0.1 M KOH	Angew. Chem. Int. Ed. 2014, 53, 7281
g-C_3N_4–GO	Powder	539	69	0.1 M KOH	ChemSusChem 2014, 7, 2125
echo-MWCNTs	Powder	360	41	1 M KOH	J. Am. Chem. Soc. 2015, 137, 2901
N-GRW	Powder	360	47	1 M KOH	Sci. Adv. 2016; 2 : e1501122
N,P,F-doped GO	Powder	390	136	0.1 M KOH	Angew. Chem. 2016, 128, 13490
O-CNT	Powder	360	48	1 M KOH	ACS Energy Lett. 2017, 2, 294
PDDA@CNTs93	Powder	357	76	1 M KOH	Energy Environ. Sci. 2018, 11, 3334
B, N-C	Powder	380	84	1 M KOH	Adv. Sci. 2018, 5, 1800036
pyridinic-N-doped graphene	Powder	450	132	1 M KOH	ACS Energy Lett. 2018, 3, 1183
COF@CNT	Powder	389	101	0.1 M KOH	ACS Nano 2021, 15, 2, 3309

Supplementary Table 3. Calculated vibrational wavenumbers of *O-O on the carboxylate group

Electrolyte	$\nu_{O\text{-}O}$ (cm^{-1})
^{16}O-KOH	998
^{18}O-KOH	951

Supplementary Table 4. The correction of zero point energy (ZPE) and entropy of the intermediates on the left/middle/right carboxylate carbon site of bare PANa and those on the left carboxylate carbon side of graphene-supported PANa.

	ZPE (eV)	TS(eV)
H_2O	0.57/0.57/0.57/0.57	0.67/0.67/0.67/0.67
H_2	0.27/0.27/0.27/0.27	0.41/0.41/0.41/0.41
*OH	0.51/0.51/0.51/0.48	0.03/0.02/0.03/0.02
*O	0.25/0.25/0.25/0.22	0.01/0.01/0.01/0.01
*OOH	0.47/0.49/0.45/0.51	0.12/0.08/0.16/0.07

Note: the values of the left, middle, and right carboxylate carbon sites of bare PANa are given in red, orange, and green colours, while the values of the left site with graphene support are in blue.

Supplementary References

1. Surendranath, Y., Nocera, D. G. Oxygen Evolution Reaction Chemistry of Oxide-Based Electrodes. *Prog. Inorg. Chem.* **57**, 505-560 (2011).

2. Li, L. et al. Unraveling Oxygen Evolution Reaction on Carbon-Based Electrocatalysts: Effect of Oxygen Doping on Adsorption of Oxygenated Intermediates. *ACS Energy Lett.* **2**, 294–300 (2017).

3. Norjmaa, G. et al. Beyond Continuum Solvent Models in Computational Homogeneous Catalysis. *Top. Catal*. **65**, 118-140 (2022).

4. Chen, X. et al. Molecular modeling of temperature dependence of solubility parameters for amorphous polymers. *J. Mol. Model.* **18**, 2333-2341 (2012).

5. Lu, L. et al. Insight into the anti-aging mechanisms of natural phenolic antioxidants in natural rubber composites using a screening strategy based on molecular simulation. *RSC Adv.* **10***,* 21318-21327 (2020).

6. Fifen, J. J., Agmon, N. Structure and spectroscopy of hydrated sodium ions at different temperatures and the cluster stability rules. *J. Chem. Theory Comput.* **12***,* 1656-1673 (2016).

7. Wang, P. et al. Hydrated Sodium Ion Clusters [$Na^+(H_2O)_n$ (n= 1–6)]: An ab initio Study on Structures and Non-covalent Interaction. *Front. Chem.* **7***,* 624-634 (2019).

8. Zhao, X., Liu, Y. Unveiling the Active Structure of Single Nickel Atom Catalysis: Critical Roles of Charge Capacity and Hydrogen Bonding. *J. Am. Chem. Soc.* **142**, 5773–5777 (2020).

9. Zhao, X., Liu, Y. Origin of Selective Production of Hydrogen Peroxide by Electrochemical Oxygen Reduction. *J. Am. Chem. Soc.* **143**, 9423–9428 (2021).

10. Hao, Q. et al., Nickel dual-atom sites for electrochemical carbon dioxide reduction. *Nat. Synthesis* **1**, 719-728 (2022).

11. Sun, K. et al., Interfacial water engineering boosts neutral water reduction. *Nat. Commun*. **13**, 6260 (2022).

12. Trotochaud, L. et al. Nickel–iron oxyhydroxide oxygen-evolution electrocatalysts: the role of intentional and incidental iron incorporation. *J. Am. Chem. Soc.* **136**, 6744-6753 (2014).

13. Wang, L., Ambrosi A. & Pumera M. “Metal-free” catalytic oxygen reduction reaction on heteroatom-doped graphene is caused by trace metal impurities. *Angew. Chem.* **125**, 14063-14066 (2013).

14. Zhang, B. et al., Homogeneously dispersed multimetal oxygen-evolving catalysts. *Science* **352**, 333-337 (2016).

15. Xu, L. et al. Plasma-engraved Co_3O_4 nanosheets with oxygen vacancies and high surface area for the oxygen evolution reaction, *Angew. Chem.* **128**, 5363-5367 (2016).

16. Zhao, S. et al. Ultrathin metal–organic framework nanosheets for electrocatalytic oxygen evolution, *Nat. Energy*, **1**, 16184 (2016).

17. Bernt, M. et al. Effect of the IrO_x conductivity on the anode electrode/porous transport layer interfacial resistance in PEM water electrolyzers, *J. Electrochem. Soc.* **168***,* 084513-084523 (2021).

18. Chow, K. F., Carducci, T. M., & Murray, R. W. Electronic conductivity of films of electroflocculated 2 nm iridium oxide nanoparticles, *J. Am. Chem. Soc.* **136***,* 3385-3387 (2014).

19. Kim, Y. T. et al. Balancing activity, stability and conductivity of nanoporous core-shell iridium/iridium oxide oxygen evolution catalysts, *Nat. Commun.* **8**, 1449 (2017).

20. Zhang, J. et al. A metal-free bifunctional electrocatalyst for oxygen reduction and oxygen evolution reactions. *Nat. Nanotech.* **10**, 444-452 (2015)

21. Yang, H. et al. Identification of catalytic sites for oxygen reduction and oxygen evolution in N-doped graphene materials: Development of highly efficient metal-free bifunctional electrocatalyst. *Sci. Adv.* **2**, e1501122 (2016)

22. Gong, K. et al. Nitrogen-Doped Carbon Nanotube Arrays with High Electrocatalytic Activity for Oxygen Reduction. *Science*, **323**, 760-764 (2009).

23. Liang, J. et al. Sulfur and Nitrogen Dual-Doped Mesoporous Graphene Electrocatalyst for Oxygen Reduction with Synergistically Enhanced Performance. *Angew. Chem. Int. Ed.* **51**, 11496-11500 (2012).

24. Ma, T. et al. Phosphorus-Doped Graphitic Carbon Nitrides Grown In Situ on Carbon-Fiber Paper: Flexible and Reversible Oxygen Electrodes. *Angew. Chem. Int. Ed.* **54**, 4646-4650 (2015).

25. Moysiadou, A. et al. Mechanism of Oxygen Evolution Catalyzed by Cobalt Oxyhydroxide: Cobalt Superoxide Species as a Key Intermediate and Dioxygen Release as a Rate-Determining Step. *J. Am. Chem. Soc.* **142**, 11901–11914 (2020).
