# Supplementary material for: A polymeric hydrogel electrocatalyst for direct water oxidation
Source: Nat Commun. 2023 Feb 13;14:818. doi: 10.1038/s41467-023-36532-x (PMC9925792; doi:10.1038/s41467-023-36532-x)
Supplement: Supplementary file 2 — Description of Additional Supplementary Files [file 41467_2023_36532_MOESM2_ESM.docx]

Description of Additional Supplementary Files

**Supplementary Video 1**

A movie showing that the bare carbon cloth electrode is hydrophobic with a large contact angle.

**Supplementary Video 2**

A movie showing that the CC-PANa electrode is superhydrophilic with an extremely small contact angle.
